# Exploring Chemical Composition of the Aerial Parts of *Vernoniastrum migeodii* and Anti-Inflammatory Activity of the Compounds

**DOI:** 10.3390/plants15020321

**Published:** 2026-01-21

**Authors:** Morteza Yazdani, Dóra Paróczai, Anita Barta, Katalin Burián, Judit Hohmann

**Affiliations:** 1 HUN-REN–USZ Biologically Active Natural Products Research Group, University of Szeged, Eötvös Str. 6, 6720 Szeged, Hungary; morteza.yazdani@szte.hu (M.Y.); barta.anita@szte.hu (A.B.); 2 Department of Medical Microbiology, Albert Szent-Györgyi Health Center and Albert Szent-Györgyi Medical School, University of Szeged, Dóm Square 10, 6720 Szeged, Hungary; paroczai.dora@med.u-szeged.hu (D.P.); burian.katalin@med.u-szeged.hu (K.B.); 3 Department of Pulmonology, Faculty of Medicine, University of Szeged, Alkotmány Str. 36, 6772 Deszk, Hungary; 4 Department of Pharmacognosy, University of Szeged, Eötvös Str. 6, 6720 Szeged, Hungary

**Keywords:** *Vernoniastrum migeodii*, Asteraceae, germacranolide sesquiterpenes, anti-inflammatory activity

## Abstract

Therapeutic strategies that fine-tune epithelial inflammatory responses are highly sought after in respiratory and mucosal disorders, but few molecules selectively target these pathways. *Vernoniastrum migeodii* (S. Moore) Isawumi (Asteraceae) represents a chemically promising but understudied source of bioactive small molecules. This study aimed to define the metabolite profile of *V*. *migeodii* and evaluate the modulation of inflammatory epithelial signaling of the constituents. From the methanolic extract of *V*. *migeodii*, five germacranolide sesquiterpenes, vernolide (**1**), 3′-hydroxylvernolide (**2**), pectorolide (**3**), 4′-hydroxypectorolide-14-*O*-acetate (**4**) and 4′-hydroxypectorolide (**5**), together with (6*S*,9*R*)-vomifoliol (**6**), eucarvone (**7**), luteolin (**8**), and luteolin-7-*O*-glucoside (**9**) were isolated by multiple chromatographic separations. The structures were determined by comprehensive 1D and 2D NMR spectroscopy. Isolated compounds **1** to **9** together with previously reported steroids (**10**–**17**) and tripeptide (**18**) were evaluated in LPS-activated A549 cells by quantitative PCR for interleukin-6 (*IL6*), interleukin-1β (*IL1β*), and prostaglandin-endoperoxide synthase 2 (*PTGS2*) and by enzyme-linked immunosorbent assay (ELISA) for IL-6 and IL-8. Compounds **2**, **7**, steroids **10**–**17** and aurantiamide acetate (**18**) reduced *IL6* mRNA relative to the LPS control, while (6*S*,9*R*)-vomifoliol (**6**) increased IL-6 and elevated IL-8. In the assay *IL1β* and *PTGS2* transcripts were not significantly altered. These findings highlight the potential of *V*. *migeodii* metabolites as modulators of epithelial inflammatory pathways. Combining chemical and biological evidence provides a clear basis for structure–activity- and pathway-focused studies.

## 1. Introduction

Inflammation is a fundamental defense response, yet when dysregulated it contributes to arthritis, cardiovascular disease, neurodegeneration, and cancer [1]. Macrophages are central effectors; after lipopolysaccharide (LPS) stimulation, they release mediators and cytokines, including nitric oxide, tumor necrosis factor alpha (TNF-α), interleukin 1βa (*IL1β*), and interleukin 6 (IL-6), which amplify innate immune signaling. Sustained overproduction of these pro-inflammatory factors damages healthy tissues and promotes chronic inflammatory disorders. Consequently, attenuation of macrophage-derived mediators is a rational path to disease modification [2,3]. In this context, natural products provide anti-inflammatory compounds with tractable mechanism of action, motivating the discovery and characterization of new bioactive metabolites [4,5,6].

In the literature, numerous examples can be found in which sesquiterpenes are efficient in anti-inflammatory test models [7,8,9]. Sesquiterpene lactones (SLs) constitute a broad and chemically diverse class of secondary metabolites of plants distributed in numerous families, Asteraceae representing the richest source in terms of the number of reported structures [10,11,12]. The anti-inflammatory activity of sesquiterpenes, particularly sesquiterpene lactones (SLs), is closely related to their α,β-unsaturated-γ-lactone moiety. SL, 3-epi-leptocarpin, isolated from *Campovassouria cruciate* (Vell.) R.M.King & H.Rob., significantly inhibited IL-6 and IL-8 release and ROS production in A549 cells previously stimulated with IFN-γ and TNF-α [13]. *Cichorium intybus* L. (Asteraceae, Cichorieae) has a well-documented history of culinary and medicinal use. In RAW264.7 macrophages stimulated by LPS, its SLs potently inhibited nitric oxide production (IC_50_ 1.83–38.81 μM) and significantly decreased the secretion of pro-inflammatory cytokines, including *TNF-α* and *IL6* [14]. Sesquiterpene lactones of the germacranolide type were identified from *Carpesium divaricatum* Siebold & Zucc., a traditional remedy used for inflammatory conditions. At subcytotoxic concentrations (0.5–2.5 µM), these compounds significantly and dose-dependently reduced the secretion of pro-inflammatory cytokines/chemokines in LPS-stimulated human neutrophils [15]. In LPS-stimulated RAW264.7 macrophages, germacranolide sesquiterpenes from *Carpesium lipskyi* C.Winkl. significantly inhibited NO production and reduced iNOS and COX-2 expression. Mechanistic assays implicated suppression of NF-κB/MAPK signaling and activation of the Nrf2/HO-1 pathway, consistent with the traditional antipyretic and anti-inflammatory uses of the plant [16]. Germacranolide SLs of the Caribbean medicinal plant *Neurolaena lobata* (L.) Cass. inhibited LPS- and TNF-α-induced upregulation of the pro-inflammatory molecules E-selectin and interleukin-8 in HUVECtert and THP-1 cells [17].

*Vernoniastrum migeodii* (S. Moore) Isawumi (syn. *Vernonia migeodii* S. Moore; *Vernonia courtetii* O. Hoffm. & Muschl.) (Asteraceae) is documented in ethnobotanical surveys in Southeast Nigeria as a traditional antimalarial remedy; the aerial parts are typically soaked in water and left to ferment before use [18]. As part of our ongoing investigation of bioactive plant metabolites, *V. migeodii* compounds were examined. In our previous work, four previously undescribed steroids, vernomigeodiins A–D (**10**–**13**) were isolated, together with known sterols (**14**–**17**) and the tripeptide aurantiamide acetate (**18**) from the chloroform extract of *V*. *migeodii* [19]. The antiviral activity of the isolated compounds against herpes simplex virus type 2 was assessed and the interactions between the ligand and the target were explored by molecular docking [19]. Despite extensive reporting of anti-inflammatory natural products, progress is often constrained by variability between assay systems and incomplete mechanistic annotation. This highlights the need for rigorously defined metabolites supported by robust structural elucidation and standardized bioactivity profiling [20,21].

The present study describes the isolation and structural characterization of additional compounds and expands the assessment of their biological activities, further clarifying the chemical profile and pharmacological potential of *V. migeodii*.

## 2. Results

### 2.1. Compound Identification

**Figure 1:**
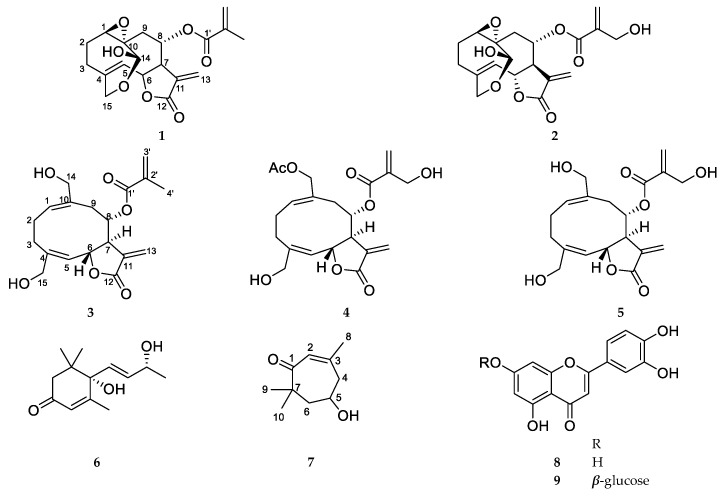

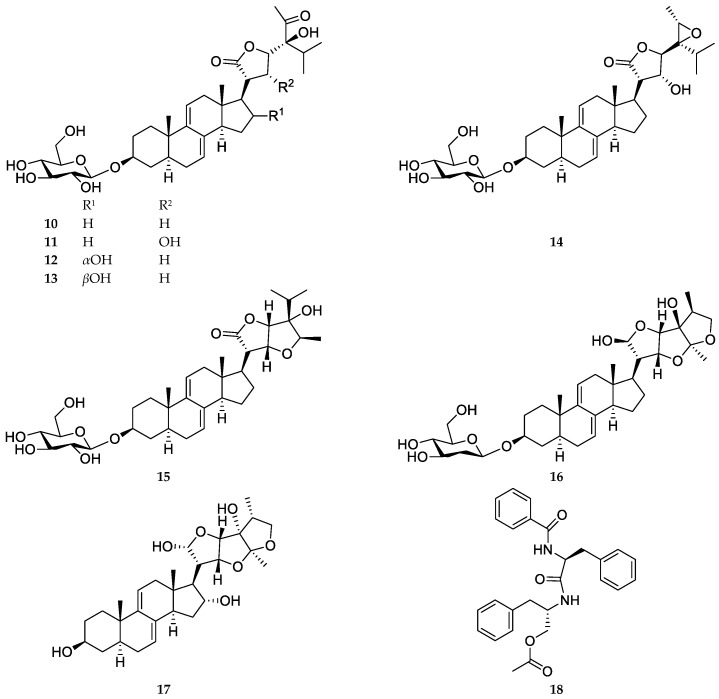
Structure of *Vernoniastrum migeodii* metabolites **1**–**18**.

Five sesquiterpenes, vernolide (**1**), 3′-hydroxyvernolide (**2**), pectorolide (**3**), 4′-hydroxypectorolide-14-*O*-acetate (**4**), and 4′-hydroxypectorolide (**5**) were isolated from the aerial parts of *V. migeodii*, along with megastigmane (6*S*,9*R*)-vomifoliol (**6**), monoterpene eucarvone (**7**) and flavones luteolin (**8**) and luteolin-7-*O*-glucoside (**9**) (Figure 1). All compounds were described for the first time from this species. The sesquiterpenes are based on germacranolide skeleton, esterified with methacrylic (**1**, **3**), hydroxymethacrylic (**2**, **4**, **5**) and acetic acids (**4**). Vernolide (**1**) and 3′-hydroxyvernolide (**2**) contain an 1,10-epoxy group and an unusual oxygen bridge between C-14 and C-15. The structure of the compounds was elucidated using 1D (^1^H NMR and ^13^C NMR JMOD) and 2D NMR (^1^H-^1^H COSY, NOESY, HSQC, HMBC) measurements. NMR data were in good agreement with those published for vernolide (**1**) [22], pectorolide (**3**) [23], 4′-hydroxypectorolide-14-*O*-acetate (**4**) [24], 4′-hydroxypectorolide (**5**) [25], (6*S*,9*R*)-vomifoliol (**6**) [26], eucarvone (**7**) [27], luteolin (**8**) and luteolin-7-*O*-glucoside (**9**) [28]. Our two-dimensional NMR studies allowed assignment of NMR signals in solvents other than published for vernolide (**1**) and 4′-hydroxypectorolide (**5**), and determination of unpublished NMR data for 3′-hydroxyvernolide (**2**), pectorolide (**3**), and eucarvone (**7**) (Tables S1 and S2).

### 2.2. Anti-Inflammatory Assay

A549 cells were treated simultaneously (co-treatment) with LPS (5 μg/mL) and the test compounds and incubated for 72 h. Under these conditions, LPS induced a robust inflammatory response, reflected by a marked increase in IL-8 secretion compared with untreated controls. Cell viability remained ≥90% at the tested compound concentrations (MTT pre-screen), confirming that the assay conditions were suitable for evaluating anti-inflammatory effects under non-cytotoxic conditions.

In this LPS-stimulated A549 co-treatment model, compounds **1**–**7** together with the previously reported constituents (**10**–**18**) were evaluated for anti-inflammatory activity by quantifying IL-8 secretion after 72 h. The structures of compounds **10**–**18** are shown in Figure 1: vernomigeodiin A–C (**10**–**12**), vernoamyoside D (**13**), vernonioside B_1_ (**14**), vernoamyoside E (**15**), vernoamyoside B (**16**), (3*β*,5*α*,16*α*,21*R*,22*R*,23*S*,25*R*,28*S*)-21,23:22,28:25,28-triepoxy-3,16,21,24-tetrahydroxystigmasta-7,9(11)-diene (**17**), and aurantiamide acetate (**18**) [19].

### 2.3. Gene Expression Analysis by qPCR

**Figure 2:**
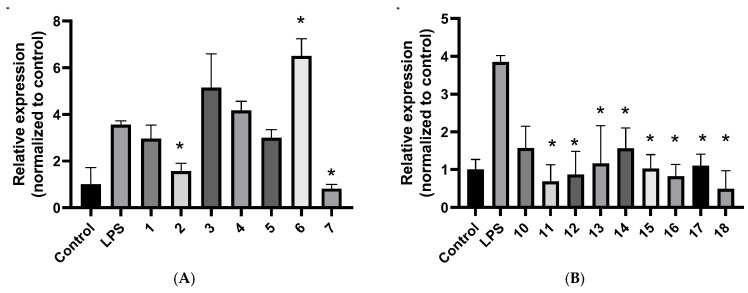
*IL6* mRNA in LPS-stimulated A549 cells measured by real-time qPCR. (**A**): compounds **1**–**7** at 10 μM. (**B**): compounds **10**–**17** at 5 μM and **18** at 10 μM. Data are presented as mean ± SD (n = 3). * *p* < 0.05.

Real-time quantitative PCR analysis demonstrated that several compounds reduced *IL6* mRNA expression in LPS-stimulated A549 cells. Relative expression was normalized to the untreated control (control = 1). In A549 cells stimulated with LPS [LPS only relative gene expression (Rge) = 3.564 ± 0.163, n = 3], real-time quantitative PCR showed that at 10 µM concentration, 3′-hydroxyvernolide (**2**) (Rge 1.569 ± 0.338), eucarvone (**7**) (Rge 0.814 ± 0.189), markedly suppressed *IL6* mRNA levels, while (6*S*,9*R*)-vomifoliol (**6**) (Rge 6.498 ± 0.749) induced an increase in *IL6* expression (Figure 2A).

In a separate set at 5 µM concentration (LPS only Rge = 3.855 ± 0.163, n = 3), the steroids vernomigeodiin A (**10**) (Rge 1.573 ± 0.577), vernomigeodiin B (**11**) (Rge 0.686 ± 0.443), vernomigeodiin C (**12**) (Rge 0.867 ± 0.616), vernoamyoside D (**13**) (Rge 1.159 ± 1.002), vernonioside B_1_ (**14**) (Rge 1.562 ± 0.537), vernoamyoside E (**15**) (Rge 1.028 ± 0.368), vernoamyoside B (**16**) (Rge 0.822 ± 0.312), and (3*β*,5*α*,16*α*,21*R*,22*R*,23*S*,25*R*,28*S*)-21,23:22,28:25,28-triepoxy-3,16,21,24-tetrahyroxy-stigmasta-7,9(11)-diene (**17**) (Rge 1.102 ± 0.308) and aurantiamide acetate (**18**) (Rge 0.493 ± 0.474) produced robust reductions in *IL6* relative to LPS (Figure 2B).

For other inflammatory markers like *IL1β* and *PTGS2* (COX-2), no consistent changes were observed under the conditions tested and the results did not show significant differences with the positive control cells stimulated with LPS (Figures S2 and S3). According to literature data, *IL1β* is only weakly or not up-regulated at all in A549 cells in response to classical LPS or cytokine stimulation [29].

### 2.4. Cytokine Measurements by ELISA

**Figure 3:**
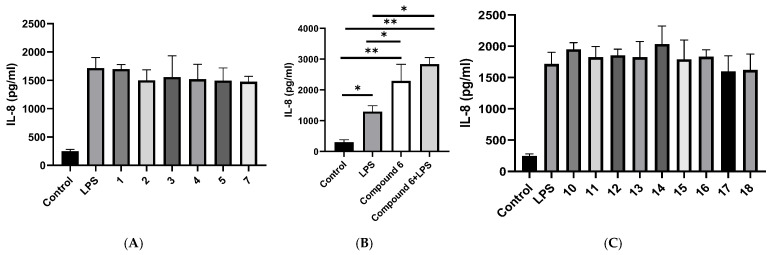
IL-8 protein in culture supernatants measured by ELISA at 72 h. (**A**) IL-8 secretion in A549 cells treated with LPS and compounds **1**–**5**, **7**, and 18 at 10 μM; (**B**) Effect of (6*S*,9*R*)-vomifoliol (**6**) at 10 μM on IL-8 production in the absence and presence of LPS; (**C**) IL-8 secretion in A549 cells treated with LPS and compounds **10**–**17** at 5 μM; Data are presented as mean ± SD of n independent experiments performed in triplicate. * *p* < 0.05, ** *p* < 0.01.

In culture supernatants collected at 72 h, IL-6 concentrations were similarly low in all groups, without detectable differences; therefore, statistical comparisons were not performed (Figure S4). On the contrary, IL-8 was quantifiable at all time points. LPS stimulation significantly increased IL-8 secretion in A549 cells compared with untreated controls at 72 h (1295.5 ± 193.8 vs. 303.5 ± 79.8 pg/mL). None of the tested compounds reduced IL-8 production (Figure 3A,C). In contrast, (6*S*,9*R*)-vomifoliol (**6**, 10 μM) significantly increased IL-8 secretion in the absence of LPS (2292.6 ± 542.1 pg/mL) and further enhanced cytokine production in the presence of LPS (2834.3 ± 219.2 pg/mL), resulting in significantly higher IL-8 levels compared with LPS treatment alone (Figure 3B). ELISA measurements were repeated in two independent experiments, each performed in triplicates with consistent results.

## 3. Discussion

This study expands the phytochemical knowledge on *V*. *migeodii* by reporting nine constituents, five germacranolide-type sesquiterpenes (**1**–**5**), megastigmane (6*S*,9*R*)-vomifoliol (**6**), monoterpene eucarvone (**7**), and flavones luteolin (**8**) and luteolin-7-*O*-glucoside (**9**). Comprehensive 1D and 2D NMR spectroscopy was performed to elucidate the structures and determine previously unpublished ^1^H and ^13^C NMR assignments for compounds **1**–**3**, **5**, and **7** (Tables S1 and S2). The anti-inflammatory activity of the compounds reported here (**1**–**7**) and isolated earlier (**10**–**18**) was evaluated in LPS-activated A549 cells using analysis of gene expression by qPCR and cytokine measurements by ELISA.

In A549 cells, LPS elicited a robust inflammatory response, captured by increased IL-6 secretion at 72 h with preserved viability, creating an appropriately activated background for testing. Against this backdrop, selective transcriptional effects emerged. At 10 µM, 3′-hydroxyvernolide (**2**), eucarvone (**7**) and aurantiamide acetate (**18**) reduced *IL6* mRNA relative to the LPS control. In particular, the steroids vernomigeodiin A (**10**), vernomigeodiin B (**11**), vernomigeodiin C (**12**), vernoamyoside D (**13**), vernonioside B_1_ (**14**), vernoamyoside E (**15**), vernoamyoside B (**16**), and (3*β*,5*α*,16*α*,21*R*,22*R*,23*S*,25*R*,28*S*)-21,23:22,28:25,28-triepoxy-3,16,21,24-tetrahydroxystigmasta-7,9(11)-diene (**17**) exerted stronger effects at a lower concentration (at 5 µM) than the non-steroidal components, indicating that steroidal scaffolds of *V*. *migeodii* can deliver pronounced suppression of *IL6* transcription under non-cytotoxic conditions. Vernomigeodiin B (**11**) (Rge 0.686 ± 0.443) and vernoamyoside B (**16**) (Rge 0.822 ± 0.312) have two free hydroxy groups in the ring system at C-17. In contrast to the steroids, norisoprenoid C_13_ (6*S*,9*R*)-vomifoliol (**6**) behaved as a pro-inflammatory profile, increasing *IL6* transcripts and improving IL-8 at 72 h. The IL-8–elevating effect was also observed in the absence of LPS, and no significant difference was detected between vomifoliol (**6**) treatment alone and vomifoliol (**6**) + LPS co-treatment, providing internal directional contrast and supporting the selectivity of IL-6 attenuation by the other compounds. By comparison, *IL1β* and *PTGS2* mRNA levels were not significantly altered under the tested conditions (single concentration, 24 h).

The divergence between qPCR and ELISA readouts is informative rather than contradictory. Suppression of *IL6* transcripts did not translate into measurable changes in IL-6 protein because supernatant levels were below the lower limit of quantification at sampled times, while IL-8 remained robustly elevated with LPS and was not reduced by the test set. This decoupling is compatible with known differences in transcriptional kinetics, post-transcriptional control, secretion, and assay sensitivity in epithelial systems, and underscores that the dominant signal of this series is host modulation at the gene expression level rather than broad suppression of chemokines at 72 h [30,31].

In a previous report, the anti-inflammatory activity of vernoamyoside B (**16**) and vernoamyoside D (**13**) was evaluated by measuring NO production in LPS-stimulated RAW 264.7 macrophages at 50 μM. Both compounds showed weak inhibition of NO formation, with inhibition values of approximately 37.6% and 24.5%, respectively, compared to the reference inhibitor *N*-monomethyl-L-arginine at approximately 57.3% [32]. In the study by Fang et al. (2022), in vivo data reported aurantiamide acetate (**18**) as a host-directed anti-inflammatory agent in an LPS-induced acute lung injury model, with oral pretreatment associated with attenuated lung injury and decreased NF-κB and PI3K/AKT signaling [33]. Moreover, (6*S*,9*R*)-vomifoliol (**6**) was published to demonstrate a concentration-dependent ability to modulate the release of pro-inflammatory (*IL6*, *IL8*, *IL1β*, and *TNF-α*) and anti-inflammatory (IL-10) cytokines. Its impact on the *IL6*, *LOX*, *NF-κB1*, and *NF-κB2* gene expression was also evaluated in human peripheral blood mononuclear cells (PBMCs) stimulated with LPS by qRT-PCR. However, a significant effect could be detected only in >25 µM concentrations of (6*S*,9*R*)-vomifoliol (**6**) [34].

## 4. Materials and Methods

### 4.1. General Experimental Procedures

The flow injection analysis was performed with Thermo Scientific Orbitrap Exploris 240 hybrid quadrupole-Orbitrap (Thermo Fischer Scientific, Waltham, MA, USA) mass spectrometer coupled to a Waters Acquity I-Class UPLC^TM^ (Waters, Manchester, UK). A Bruker Ascend 500 spectrometer equipped with a 5-mm BBO Prodigy probe (Bruker BioSpin, Karlsruhe, Baden-Württemberg, Germany) was used to record NMR spectra in CDCl_3_ or CD_3_OD at 500 MHz for ^1^H and 125 MHz for ^13^C. Chemical shifts (δ, ppm) were referenced to residual signals of deuterated solvents. Two-dimensional NMR experiments were acquired using standard Bruker pulse programs and software, with gradient enhanced sequences for ^1^H–^1^H COSY, NOESY, HSQC, and HMBC. The acquisition parameters for the 2D NMR experiments were as follows:

^1^H-^1^H COSY: spectral width: 6578.92631 Hz (F2/F1); relaxation delay (d1): 1.354880095 s; scans: 2; increments: 1024.

NOESY: spectral width: 5000 Hz (both dimensions); mixing time: 0.3 ms; relaxation delay (d1): 1.401983857 s; scans: 4; increments: 1024.

HSQC: spectral width (F2, ^1^H): 7812.5 Hz; spectral width (F1, ^13^C): 27,668.788 Hz; relaxation delay (d1): 1.5 s; scans: 5; increments: 512.

HMBC: spectral width (F2, ^1^H): 3968.254 Hz; spectral width (F1, ^13^C): 27,669.48 Hz; optimized for long-range couplings (*J*): 8 Hz; relaxation delay (d1): 1.5 s; scans: 24; increments: 2048.

Size-exclusion (gel filtration) chromatography was used in Sephadex LH-20 (25–100 µm; Sigma-Aldrich, St. Louis, MO, USA). Vacuum liquid chromatography (VLC) was performed on silica gel (15 µm; Merck, Darmstadt, Germany) under normal phase conditions. Flash chromatography (FC) was carried out on a CombiFlash NextGen 300+ system (Teledyne ISCO, Lincoln, NE, USA) equipped with integrated UV, UV–Vis and evaporative light scattering detection, using RediSep Gold high-performance flash columns (Teledyne ISCO, Lincoln, NE, USA). The analytical and preparative HPLC separations were performed on a Shimadzu LC-20AD UFLC with a photodiode array (PDA) detector and on LC-2010CHT units with UV–Vis detection (Shimadzu, Kyoto, Japan). For reversed-phase HPLC, a LiChrospher RP-18 column (250 × 4.0 mm, 5 µm) used, while normal phase separations were performed on the LiChrospher Si 60 column (250 × 4.0 mm, 5 µm). Thin-layer chromatography (TLC) was performed on 60 F_254_ silica gel plates (Merck, Darmstadt, Germany; aluminum-backed; 0.25 mm layer thickness; 20 × 20 cm) and monitored under UV light at 254 and 366 nm, as well as after spraying with concentrated sulfuric acid followed by heating for 5 min. All solvents used for TLC were of analytical grade or higher (VWR, Debrecen, Hungary).

### 4.2. Plant Material

Samples of *Vernoniastrum migeodii* (S. Moore) Isawumi (Asteraceae) samples were collected in June 2020 in the vicinity of Zaria, Kaduna State, Nigeria (11.134179 N, 7.669629 E) and identified by the Umar Shehu Gallah National Research Institute for Chemical Technology (NARICT), Zaria, Nigeria. The voucher samples were preserved with the accession number Narict/Biores/323 at NARICT, and an additional sample (No. 899) was deposited at the Herbarium of the Department of Pharmacognosy, University of Szeged, Hungary.

### 4.3. Extraction and Isolation

The dried plant material (950 g) was crushed in a blender and then percolated with MeOH (20 L) at room temperature. After evaporation of the solvent, the dry residue (175.49 g) was dissolved in 50% aqueous MeOH and subjected to solvent–solvent partition with *n*-hexane (6 × 500 mL), chloroform (6 × 500 mL), and then ethyl acetate (6 × 500 mL). The chloroform-soluble phase (38.24 g) was fractionated by OCC on polyamide (125 g) with mixtures of MeOH-H_2_O (1:4, 2:3, 3:2, 4:1 and 5:0) as eluents to obtain MeOH fractions of 20%, 40%, 60%, 80% and 100%, respectively.

The 20% MeOH fraction (11.1 g) was purified by normal-phase flash chromatography (NP-FC) using silica gel (120 g). Initially, the column was eluted with a linear gradient of cyclohexane–EtOAc (from 20% to 80% EtOAc) over 40 min. This was followed by a second linear gradient, using a fixed cyclohexane–EtOAc mixture (1:1) combined with increasing proportions of EtOH (from 5% to 100%) for an additional 40 min. Finally, the column was eluted with 100% EtOH. Fractions exhibiting similar compositions were combined according to TLC analysis, providing fractions S1–S25 and compound **1** in crystalline form (336 mg). The purification of fraction S8 (49.5 mg) was carried out by two sequential HPLC runs of normal phase (NP-HPLC), first using an isocratic solvent system of cyclohexane-EtOAc (60:40), followed by a second run with cyclohexane-EtOAc (65:35), leading to the isolation of compounds **3** (5.48 mg), **7** (4.4 mg), and **6** (3.1 mg). The fraction S9 (83.1 mg) was chromatographed by NP-HPLC (mobile phase: cyclohexane-EtOAc 50:50), providing five subfractions (ST1–ST5). Subfraction ST2 (39.1 mg) was purified by NP-HPLC with cyclohexane-EtOAc-MeOH (55:45:8) solvent system to produce compound **4** (30.5 mg). NP-FC was applied to the separation of fraction S10 (535 mg) on silica gel (12 g) using a gradient system of cyclohexane-EtOAc (linear from 0% to 100% EtOAc, t = 50 min) and at the end eluted with MeOH (100%, t = 5 min) (SU1-SU6). Subfraction SU5 (350 mg) was then subjected to gel filtration (GF) in Sephadex LH-20 with dichloromethane–methanol (1:1) as an eluent, leading to the isolation of compound **2** (25.3 mg). Compound **5** (22.3 mg) was obtained by further purification of fraction S16 (52.4 mg) by reversed-phase HPLC (RP-HPLC) with an isocratic H_2_O-MeCN (65:35) solvent system.

The EtOAc-soluble phase (28 g) was separated by normal-phase vacuum-liquid chromatography (NP-VLC) using a gradient system of cyclohexane-EtOAc (from 100:0 to 0:100) and eluted with MeOH (100%) at the end of this process, providing compounds **8** (97 mg) and **9** (31 mg) together with additional subfractions. Details of the isolation and purification of steroids (**10**–**17**) and tripeptide (**18**) are described in our previous publication [19].

### 4.4. Spectroscopic Data of the Isolated Compounds

Vernolide (**1**): White amorphous solid; ^1^H and ^13^C NMR data: see Table S1; HRESIMS + *m*/*z* 363.1435 [M + H]^+^ (calcd for C_19_H_23_O_7_^+^ 363.1438).

3′-Hydroxyvernolide (**2**): Colorless amorphous solid; ^1^H and ^13^C NMR data: see Table S1; HRESIMS + *m*/*z* 379.1386 [M + H]^+^ (calcd for C_19_H_23_O_8_^+^ 379.1387).

4′-Hydroxypectorolide-14-*O*-acetate (**4**): Colorless amorphous solid; ^1^H and ^13^C NMR data: see Table S2; HRESIMS + *m*/*z* 429.1516 [M + Na]^+^ (calcd for C_21_H_26_O_8_Na^+^ 429.1520).

4′-Hydroxypectorolide (**5**): Colorless amorphous solid; ^1^H and ^13^C NMR data: see Table S2; HRESIMS + *m*/*z* 387.1412 [M + Na]^+^ (calcd for C_19_H_24_O_7_Na^+^ 387.1414).

Eucarvone (**7**): Colourless oily material; ^13^C NMR (125 MHz, CDCl_3_) δ (ppm): 180.8 (C-1), 113.5 (C-2), 171.6 (C-3), 48.1 (C-4), 65.3 (C-5), 50.1 (C-6), 35.2 (C-7), 25.8 (C-8), 30.1 (C-9), 25.3 (C-10).

### 4.5. Anti-Inflammatory Assay

The A549 human airway epithelial cell line was purchased from Sigma-Aldrich (Sigma-Aldrich (Merck), Darmstadt, Germany, no. 86012804). A549 cells were kept in Eagle’s Minimum Essential Medium (MEM; Sigma, St. Louis, MO, USA), supplemented with 25 μg/mL gentamicin, 10% fetal calf serum, 0.5% glucose (*w*/*v*), 0.3 mg/mL L-glutamine, and 4 mM HEPES. The cells were seeded in 6-well plates at a density of 1 × 10^6^ cells per well. Each experimental condition was performed in triplicates. Cells were treated simultaneously (co-treatment) with lipopolysaccharide (LPS, 5 μg/mL; Thermo Scientific, Waltham, MA, USA) and the test compounds and incubated for 72 h at 37 °C in a humidified atmosphere containing 5% CO_2_ before analysis. Treatment concentrations were chosen to maintain greater than 90% cell viability, as determined by the MTT assay (Figure S1) described above [35]. A549 cells were treated with compounds **1**–**7** and **18** at 10 μM and compounds **10**–**17** at 5 μM. LPS-stimulated cells served as positive controls, whereas untreated cells were regarded as negative controls.

### 4.6. mRNA Extraction and cDNA Synthesis

Total RNA was isolated from treated and control cells using TRI Reagent (Sigma-Aldrich, St. Louis, MO, USA) according to the manufacturer’s instructions. RNA concentration and purity were assessed with a NanoDrop spectrophotometer (Thermo Scientific, Waltham, MA, USA). First-strand cDNA synthesis was performed using the Maxima First-String cDNA Synthesis Kit (Thermo Fisher Scientific, Waltham, MA, USA) with 20 pM random hexamer primers, following the manufacturer’s protocol.

### 4.7. Quantitative PCR (qPCR) Validation of the Target Genes

Quantitative real-time PCR was carried out on a CFX96 system (Bio-Rad, Hercules, CA, USA) using the SensiFAST SYBR No-ROX Kit (Bioline, London, UK). Human-specific primer pairs were used as follows: β-actin (Actb) forward 5′-TTC TAC AAT GAG CTG CGT GTG GCT-3′ and reverse 5′-TAG CAC AGC CTG GAT AGC AAC GTA-3′; *PTGS2* (COX-2) forward 5′-TAC TGG AAG CCA AGC ACT TT-3′ and reverse 5′-GGA CAG CCC TTC ACG TTA TT-3′; *IL1β* forward 5′-CAA AGG CGG CCA GGA TAT AA-3′ and reverse 5′-CTA GGG ATT GAG TCC ACA TTC AG-3′; and *IL6* forward 5′-CAG CTA TGA ACT CCT TCT CCA C-3′ and reverse 5′-GCG GCT ACA TCT TTG GAA TCT-3′. Cycle threshold values (Ct) were determined for target genes and for β-actin. Relative gene expression levels were calculated using the 2^−ΔΔCt^ method, with results expressed as 2^−ΔΔCt^, where ΔΔCt = ΔCt (experimental sample) − ΔCt (control sample).

### 4.8. Cytokine Measurements

Cell culture supernatants were collected and cytokine levels were analyzed using a human IL-6 ELISA kit (Elabscience, cat. E-UNEL-H0097, Houston, TX, USA) and human IL-8 ELISA kit (Elabscience, cat. E-UNEL-H0099, Houston, TX, USA), according to the manufacturer’s instructions. The samples were analyzed in duplicate. The sensitivity range of the IL-6 assay was 1.56–100 pg/mL and for the IL-8 assay 7.81–500 pg/mL. Absorbance was measured at 450 nm with a reference wavelength of 570 nm using an EZ READ 400 ELISA reader (Biochrom, Cambridge, UK).

### 4.9. Statistical Analysis

All data are presented as mean ± standard deviation (SD) or as percentages. Statistical analysis was performed with GraphPad Prism version 8.0.1 (GraphPad Software, San Diego, CA, USA). Comparisons between groups were evaluated using one-way ANOVA or unpaired *t* tests. A *p*-value < 0.05 was considered statistically significant.

## 5. Conclusions

The present study reports new metabolites of *V. migeodii*, including esterified germacranolide sesquiterpenes (**1**–**5**), a megastigmane (**6**), a monoterpene (**7**) and widely occurring flavones (**8**, **9**). The isolated compounds were structurally characterized and investigated for possible anti-inflammatory activity in an epithelial inflammation model, together with the previously isolated compounds (**10**–**18**). In LPS stimulated A549 cells, the dominant signal was targeted attenuation of *IL6* transcripts, while *IL1β* and *PTGS2* were unchanged, and the production of the IL-8 protein was not reduced under the present conditions. Within this profile, the series of steroid (**10**–**17**) at 5 μM and aurantiamide acetate (**18**) at 10 μM showed the strongest transcriptional effects, while (6*S*,9*R*)-vomifoliol (**6**) served as a pro-inflammatory comparator, enhancing IL-8 secretion both in the absence and presence of LPS.

These results support the potential for the development of natural product scaffolds for host-directed modulation of epithelial inflammatory gene programs. By integrating different structural types with cell-based assays, the study establishes a strong foundation for future work, including time-resolved and dose-dependent analyses of *IL6* regulation, as well as mechanistic investigations of upstream transcriptional and post-transcriptional control within epithelial systems. In this context, natural product candidates that selectively modulate epithelial inflammation deserve consideration as pathway-focused modulators in situations where broad immunosuppression is not desirable.

## Data Availability

The original contributions presented in this study are included in the article/Supplementary Material. Further inquiries can be directed to the corresponding authors.

## Supplementary Materials

The following supporting information can be downloaded at: https://www.mdpi.com/article/10.3390/plants15020321/s1, Figure S1. Cell viability data for compounds **1**–**7**, and **10**–**18** tested by MTT assay on A549 cells; Figure S2. *IL1β* mRNA in LPS-stimulated A549 cells measured by real-time qPCR; Figure S3. *PTGS2* mRNA in LPS-stimulated A549 cells measured by real-time qPCR; Figure S4. IL-6 protein in culture supernatants measured by ELISA at 72 h; Figures S5–S41. ^1^H NMR, ^13^C NMR JMOD, and 2D NMR spectra of compounds **1**–**9**; Table S1. NMR data of vernolide (**1**) and 3′-hydroxyvernolide (**2**); Table S2. NMR data of pectorolide (**3**) and 4′-hydroxypectorolide (**5**).

## Abbreviations

The following abbreviations are used in this manuscript:

IL6	interleukin-6
IL1β	interleukin-1β
PTGS2	prostaglandin-endoperoxide synthase 2
ELISA	enzyme-linked immunosorbent assay
LPS	lipopolysaccharide
TNF-α	tumor necrosis factor alpha
SLs	sesquiterpene lactones
VLC	vacuum liquid chromatography
FC	flash chromatography
PDA	photodiode-array
TLC	thin-layer chromatography
NARICT	National Research Institute for Chemical Technology
NP-FC	normal-phase flash chromatography
NP-HPLC	normal-phase HPLC
GF	gel filtration
RP-HPLC	reversed-phase HPLC
NP-VLC	normal-phase vacuum-liquid chromatography
qPCR	quantitative PCR
SD	standard deviation
