# Supplementary material for: Exploring Chemical Composition of the Aerial Parts of Vernoniastrum migeodii and Anti-Inflammatory Activity of the Compounds
Source: Plants (Basel). 2026 Jan 21;15(2):321. doi: 10.3390/plants15020321 (PMC12845201; doi:10.3390/plants15020321)
Supplement: Supplementary file 1 [file plants-15-00321-s001.zip › plants-4048799-supplementary.pdf]

SUPPLEMENTARY MATERIAL

Exploring chemical composition of the aerial parts of *Vernoniastrum migeodii* and anti-inflammatory activity of the compounds

Morteza Yazdani [a], Dóra Paróczai [b c], Anita Barta [a d], Katalin Burián [b], Judit Hohmann [a d]

[a] HUN-REN–USZ Biologically Active Natural Products Research Group, University of Szeged, Eötvös Str. 6, 6720, Szeged, Hungary

[b] Department of Medical Microbiology, Albert Szent-Györgyi Health Center and Albert Szent-Györgyi Medical School, University of Szeged, Dóm Square 10, 6720, Szeged, Hungary

[c] Department of Pulmonology, Faculty of Medicine, University of Szeged, Alkotmány Str. 36, 6772, Deszk, Hungary

[d] Department of Pharmacognosy, University of Szeged, Eötvös Str. 6, 6720, Szeged, Hungary

*Correspondence: hohmann.judit@szte.hu

CONTENT

Figure S1. Cell viability data for compounds **1**–**7**, and **10**–**18** tested by MTT assay on A549 cells.4

Figure S2. IL-1β mRNA in LPS-stimulated A549 cells measured by real-time qPCR 7

Figure S3. PTGS2 mRNA in LPS-stimulated A549 cells measured by real-time qPCR 7

Figure S4. IL-6 protein in culture supernatants measured by ELISA at 72 h................................. 8

Figure S5. ^{1}H NMR spectrum of compound **1** (500 MHz, CD_3OD)... 8

Figure S6. ^{13}C NMR JMOD spectrum of compound **1** (125 MHz, CD_3OD) 9

Figure S7. HSQC spectrum of compound **1** (125/500 MHz, CD_3OD) 9

Figure S8. HMBC spectrum of compound **1** (125/500 MHz, CD_3OD).. 10

Figure S9. ^{1}H-^{1}H COSY spectrum of compound **1** (500 MHz, CD_3OD).. 10

Figure S10. NOESY spectrum of compound **1** (500 MHz, CD_3OD)... 11

Figure S11. ^{1}H NMR spectrum of compound **2** (500 MHz, $CDCl_3$)... 11

Figure S12. HSQC spectrum of compound **2** (125/500 MHz, $CDCl_3$) 12

Figure S13. HMBC spectrum of compound **2** (125/500 MHz, $CDCl_3$).. 12

Figure S14. ^{1}H-^{1}H COSY spectrum of compound **2** (500 MHz, $CDCl_3$).. 13

Figure S15. NOESY spectrum of compound **2** (500 MHz, $CDCl_3$).. 13

Figure S16. ^{1}H NMR spectrum of compound **3** (500 MHz, $CDCl_3$)... 14

Figure S17. ^{13}C NMR JMOD spectrum of compound **3** (125 MHz, $CDCl_3$) 14

Figure S18. HMBC spectrum of compound **3** (125/500 MHz, $CDCl_3$).. 15

Figure S19. ^{1}H-^{1}H COSY spectrum of compound **3** (500 MHz, $CDCl_3$).. 15

Figure S20. NOESY spectrum of compound **3** (500 MHz, $CDCl_3$).. 16

Figure S21. ^{1}H NMR spectrum of compound **4** (500 MHz, CD_3OD)... 16

Figure S22. HSQC spectrum of compound **4** (125/500 MHz, CD_3OD) 17

Figure S23. HMBC spectrum of compound **4** (125/500 MHz, CD_3OD).. 17

Figure S24. ^{1}H-^{1}H COSY spectrum of compound **4** (500 MHz, CD_3OD)....................................... 18

Figure S25. NOESY spectrum of compound **4** (500 MHz, CD_3OD) 18

Figure S26. ^{1}H NMR spectrum of compound **5** (500 MHz, CD_3OD) 19

Figure S27. HSQC spectrum of compound **5** (125/500 MHz, CD_3OD) 19

Figure S28. HMBC spectrum of compound **5** (125/500 MHz, CD_3OD) 20

Figure S29. ^{1}H-^{1}H COSY spectrum of compound **5** (500 MHz, CD_3OD) 20

Figure S30. NOESY spectrum of compound **5** (500 MHz, CD_3OD) 21

Figure S31. ^{1}H NMR spectrum of compound **6** (500 MHz, $CDCl_3$) 21

Figure S32. ^{13}C NMR JMOD spectrum of compound **6** (125 MHz, $CDCl_3$) 22

Figure S33. HSQC spectrum of compound **6** (125/500 MHz, $CDCl_3$) 22

Figure S34. HMBC spectrum of compound **6** (125/500 MHz, $CDCl_3$) 23

Figure S35. ^{1}H-^{1}H COSY spectrum of compound **6** (500 MHz, $CDCl_3$) 23

Figure S36. NOESY spectrum of compound **6** (500 MHz, $CDCl_3$) 24

Figure S37. ^{1}H NMR spectrum of compound **7** (500 MHz, $CDCl_3$) 24

Figure S38. ^{13}C NMR JMOD spectrum of compound **7** (125 MHz, $CDCl_3$) 25

Figure S39. ^{1}H NMR spectrum of compound **8** (500 MHz, DMSO-d_6) 25

Figure S40. ^{1}H NMR spectrum of compound **9** (500 MHz, DMSO-d_6) 26

Figure S41. ^{13}C NMR JMOD spectrum of compound **9** (125 MHz, DMSO-d_6) 26

Table S1. NMR data of vernolide (**1**) and 3’-hydroxyvernolide (**2**) 27

Table S2. NMR data of pectorolide (**3**) and 4’-hydroxypectorolide (**5**) 28

Compound **1**:

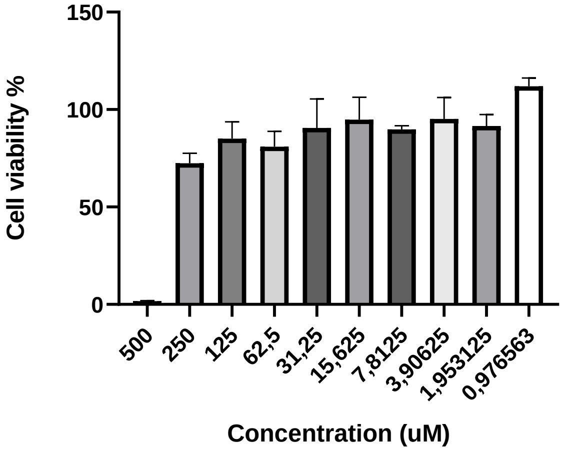

Compound **2**:

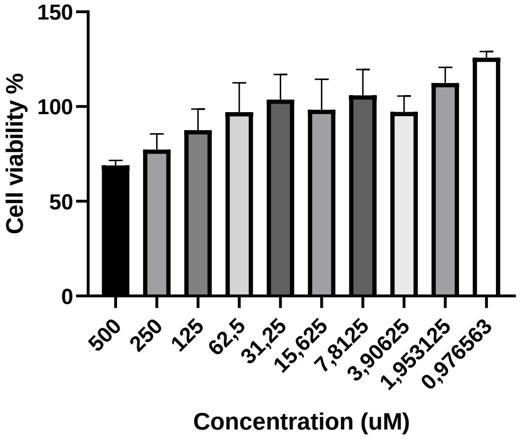

Compound **3**:

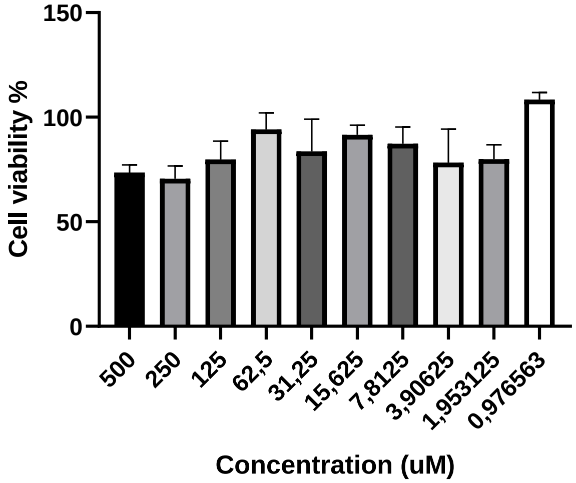

Compound **4**:

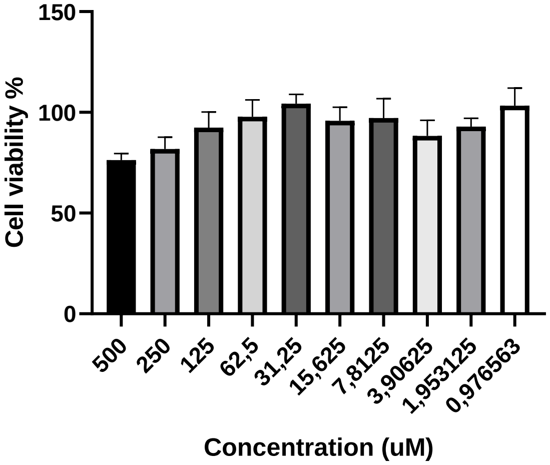

Compound **5**:

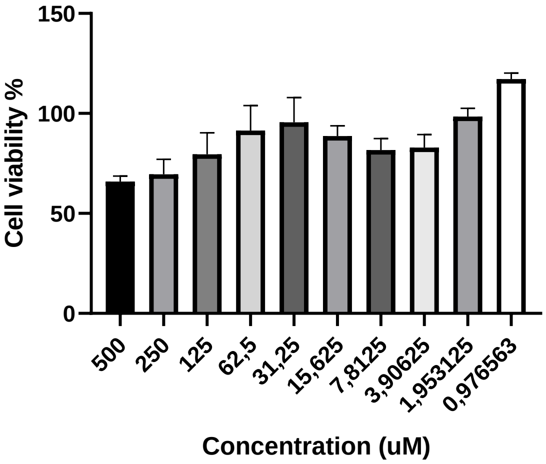

Compound **6**:

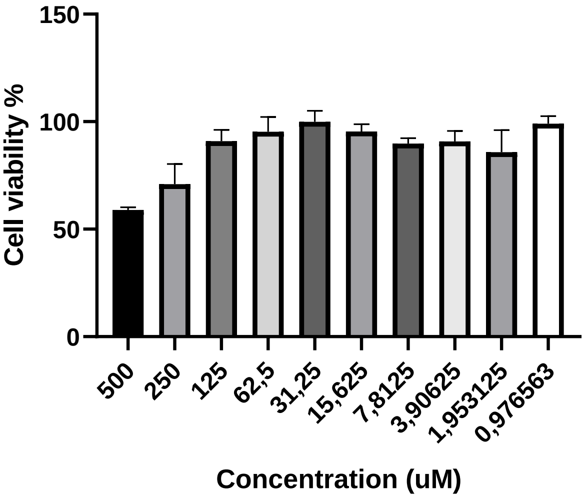

Compound **7**:

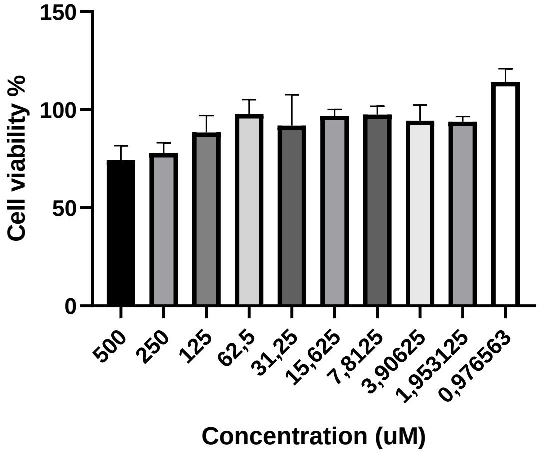

Compound **10**:

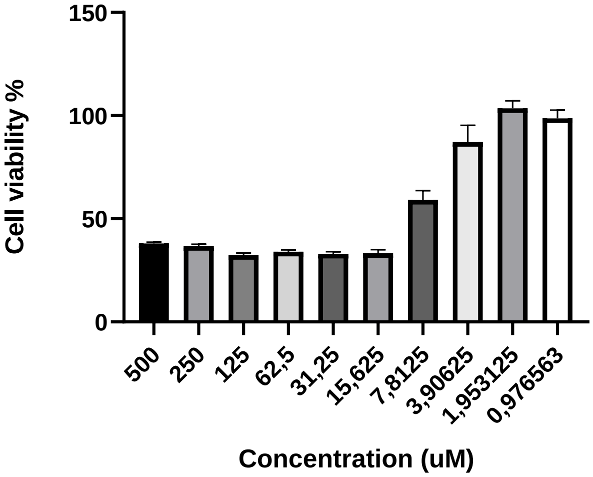

Compound **11**:

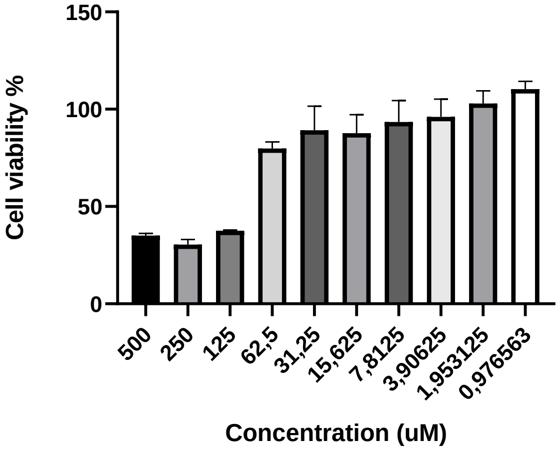

Compound **12**:

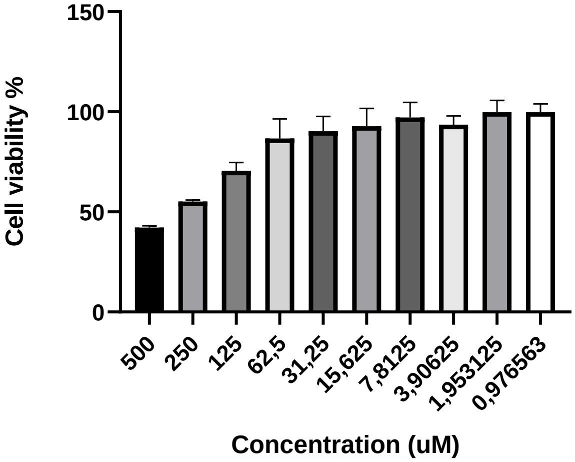

Compound **13**:

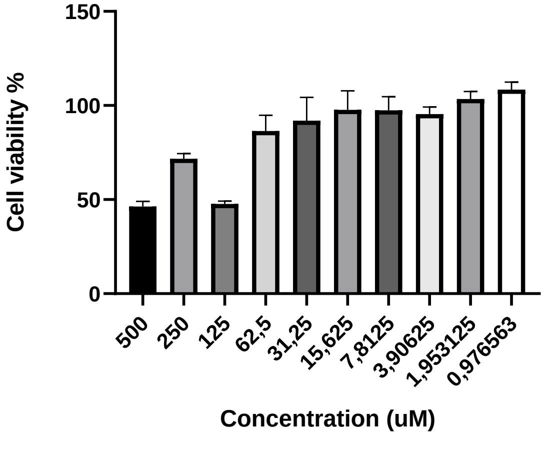

Compound **14**:

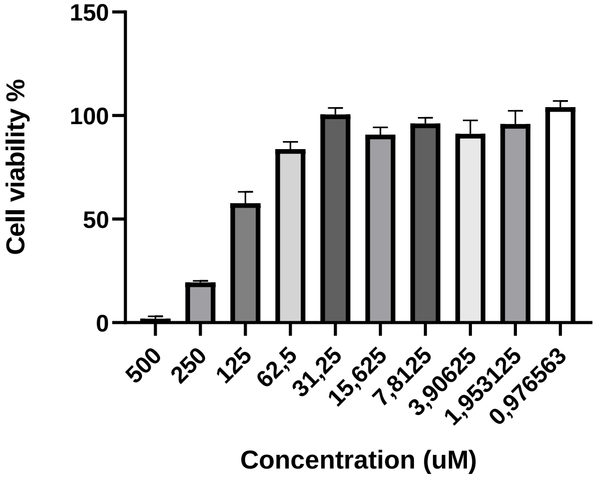

Compound **15**:

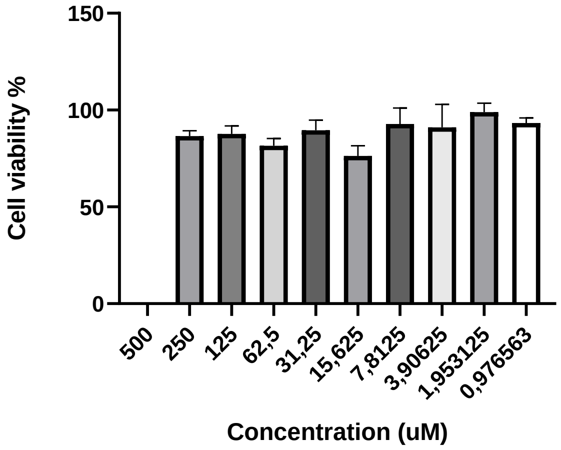

Compound **16**:

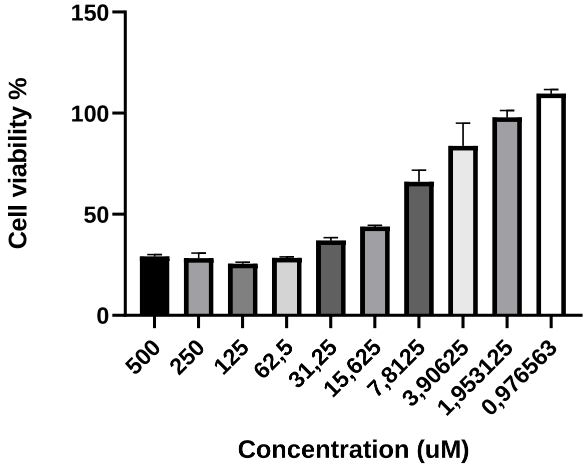

Compound **17**:

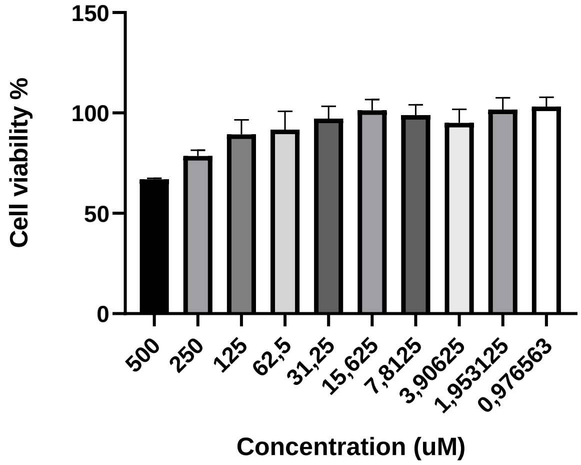

Compound **18**:

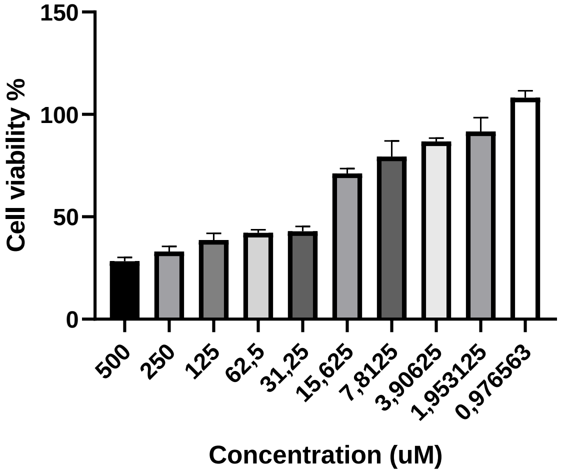

Figure S1. Cell viability data for compounds **1**–**7**, and **10**–**18** tested by MTT assay on A549 cells.

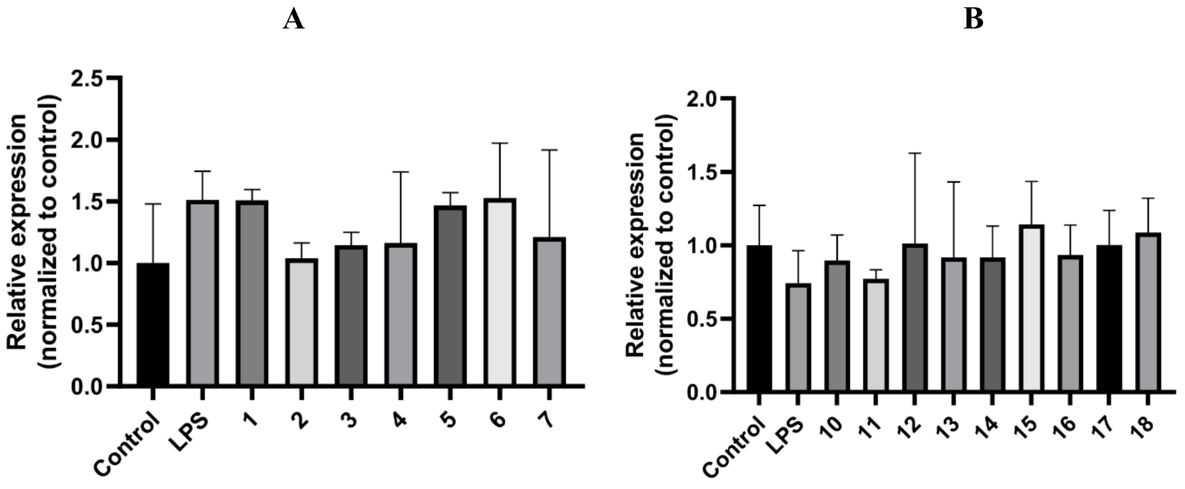

Figure S2. IL-1β mRNA in LPS-stimulated A549 cells measured by real-time qPCR. **A**: compounds **1**–**7**, and **8** at 10 μM. **B**: compounds **10**–**17** at 5 μM.

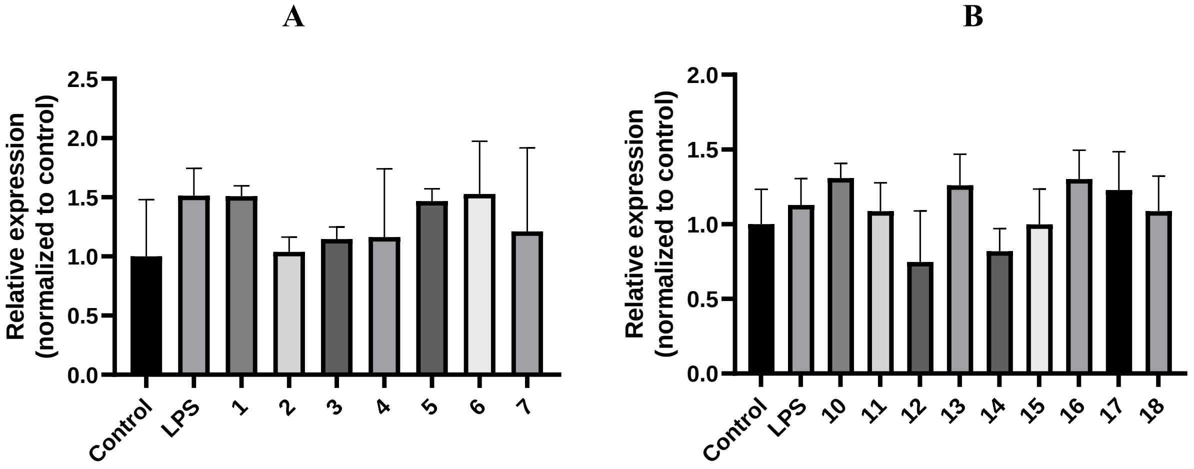

Figure S3. PTGS2 mRNA in LPS-stimulated A549 cells measured by real-time qPCR. **A**: compounds **1**–**7**, and **18** at 10 μM. **B**: compounds **10**–**17** at 5 μM.

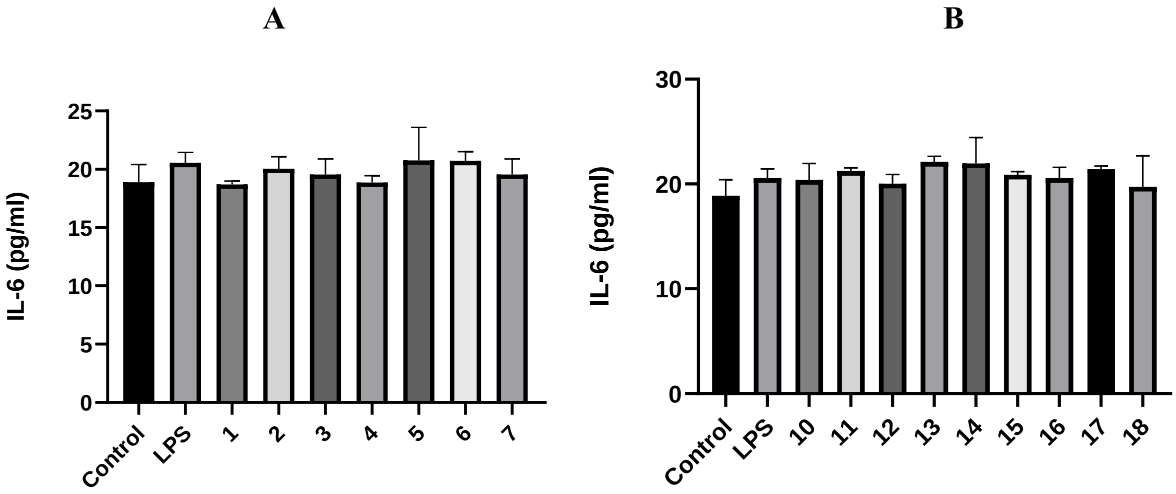

Figure S4. IL-6 protein in culture supernatants measured by ELISA at 72 h. **A**: compounds **1**–**7**, and **18** at 10 µM. **B**: compounds **10**–**17** at 5 µM.

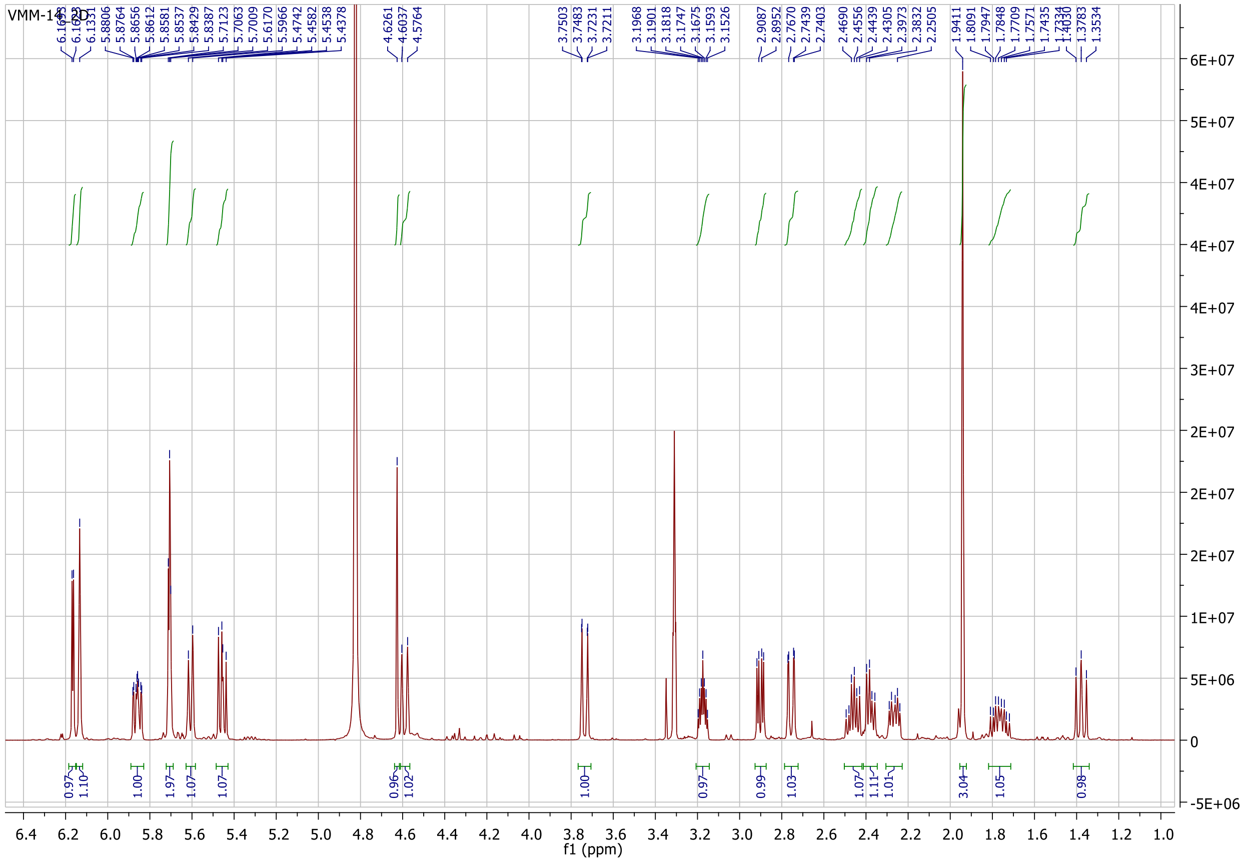

Figure S5. ^{1}H NMR spectrum of compound **1** (500 MHz, CD_3OD)

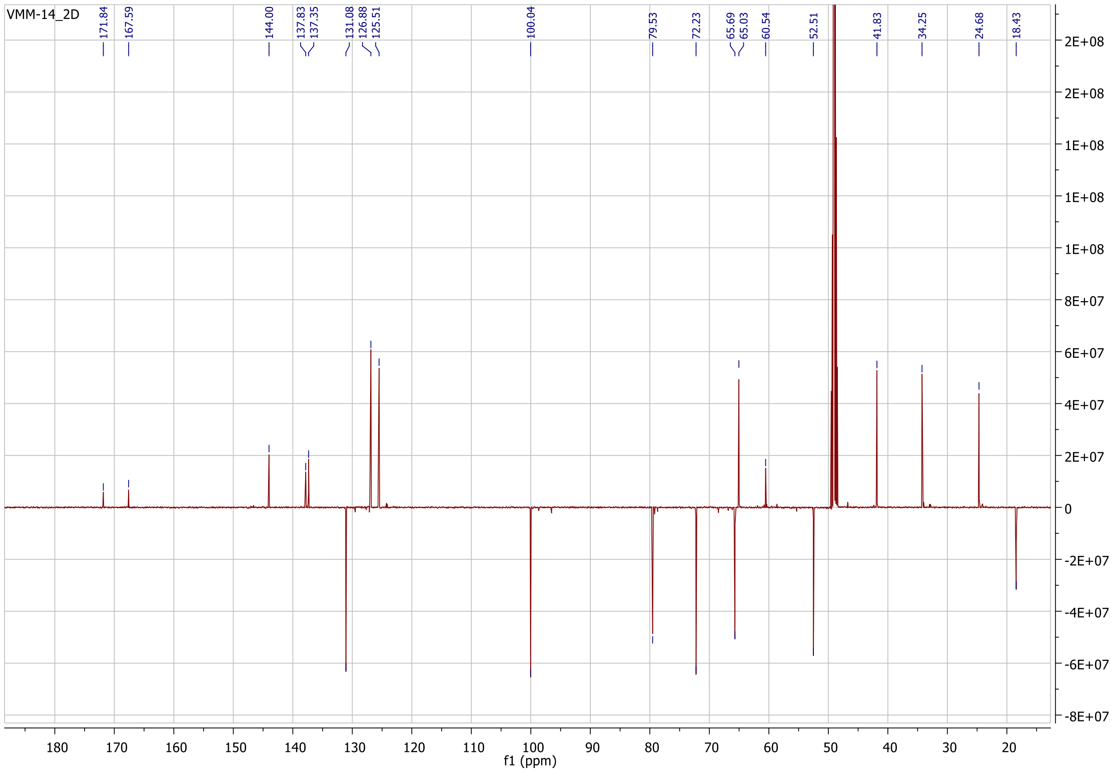

Figure S6. ^{13}C NMR JMOD spectrum of compound **1** (125 MHz, CD_3OD)

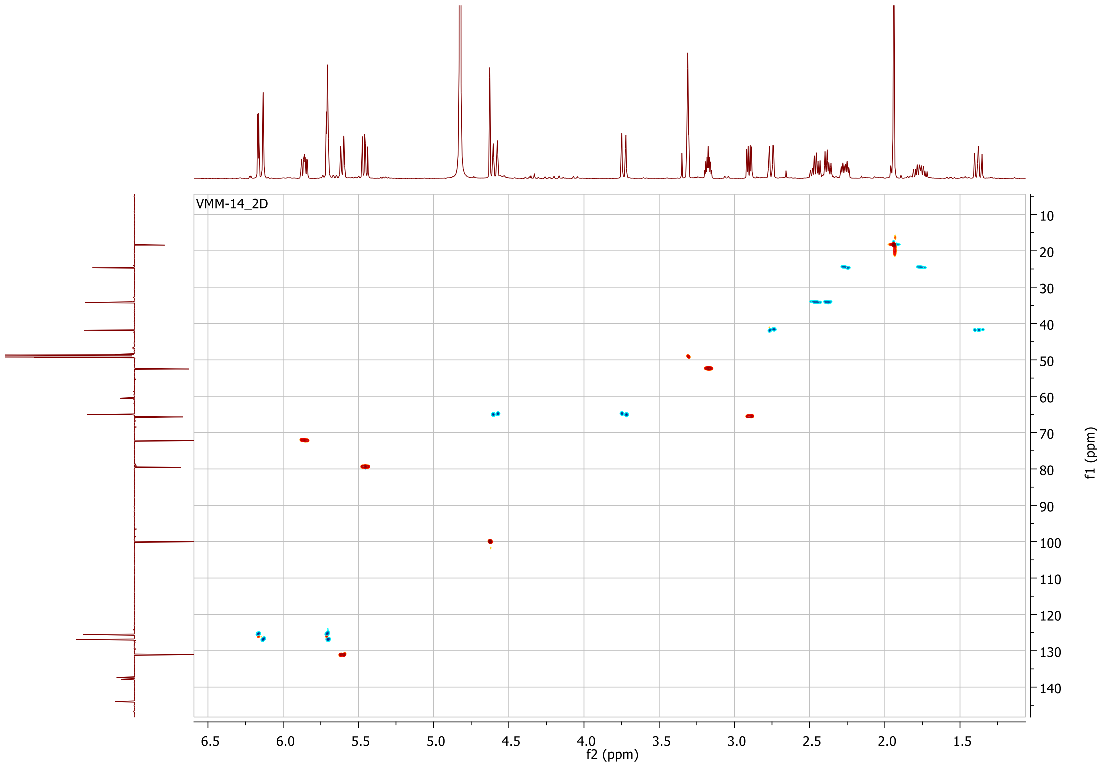

Figure S7. HSQC spectrum of compound **1** (125/500 MHz, CD_3OD)

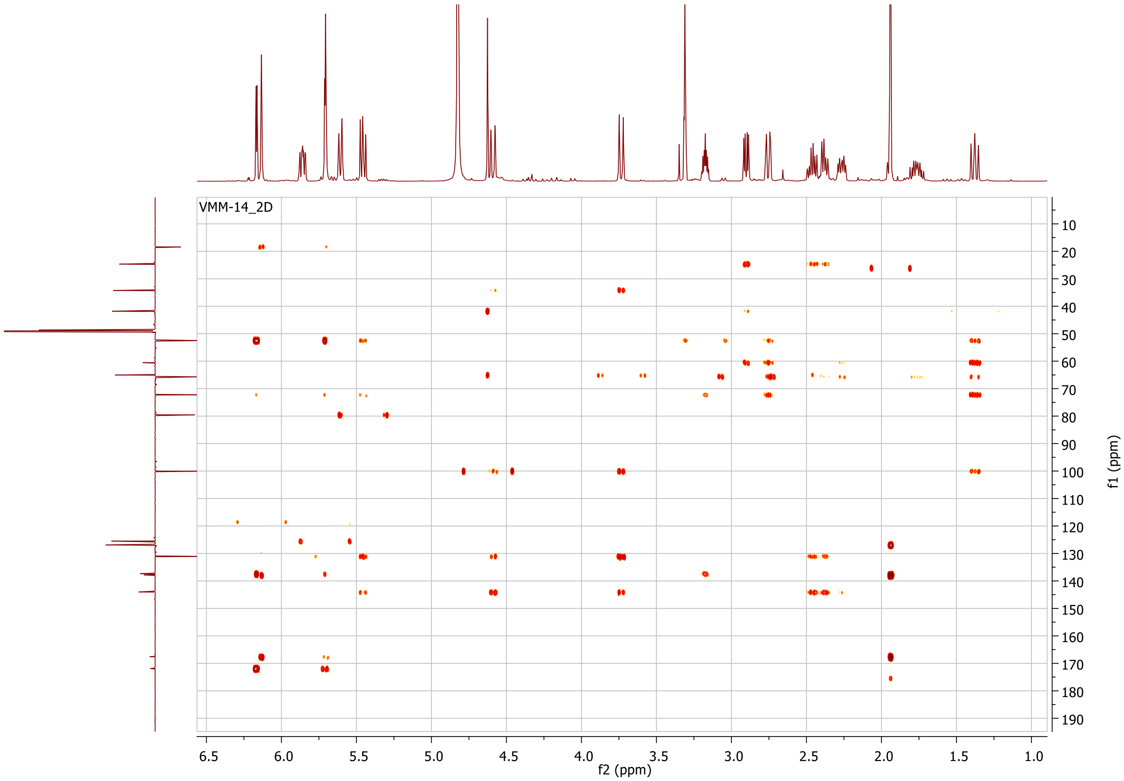

Figure S8. HMBC spectrum of compound **1** (125/500 MHz, CD_3OD)

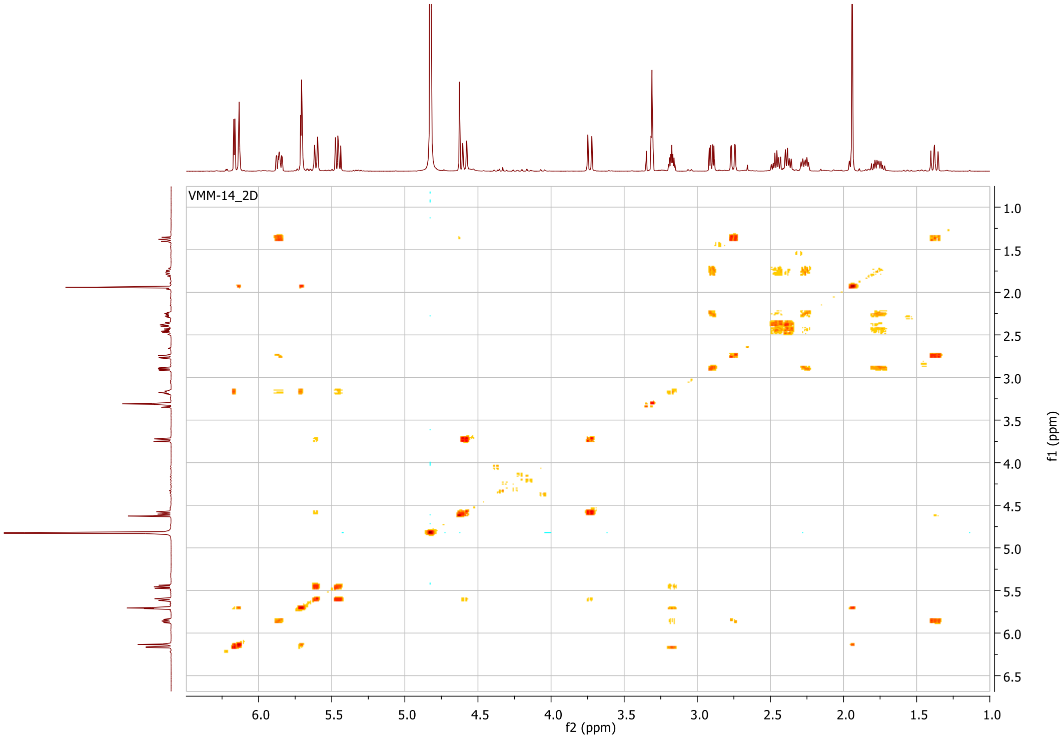

Figure S9. ^1H-^1H COSY spectrum of compound **1** (500 MHz, CD_3OD)

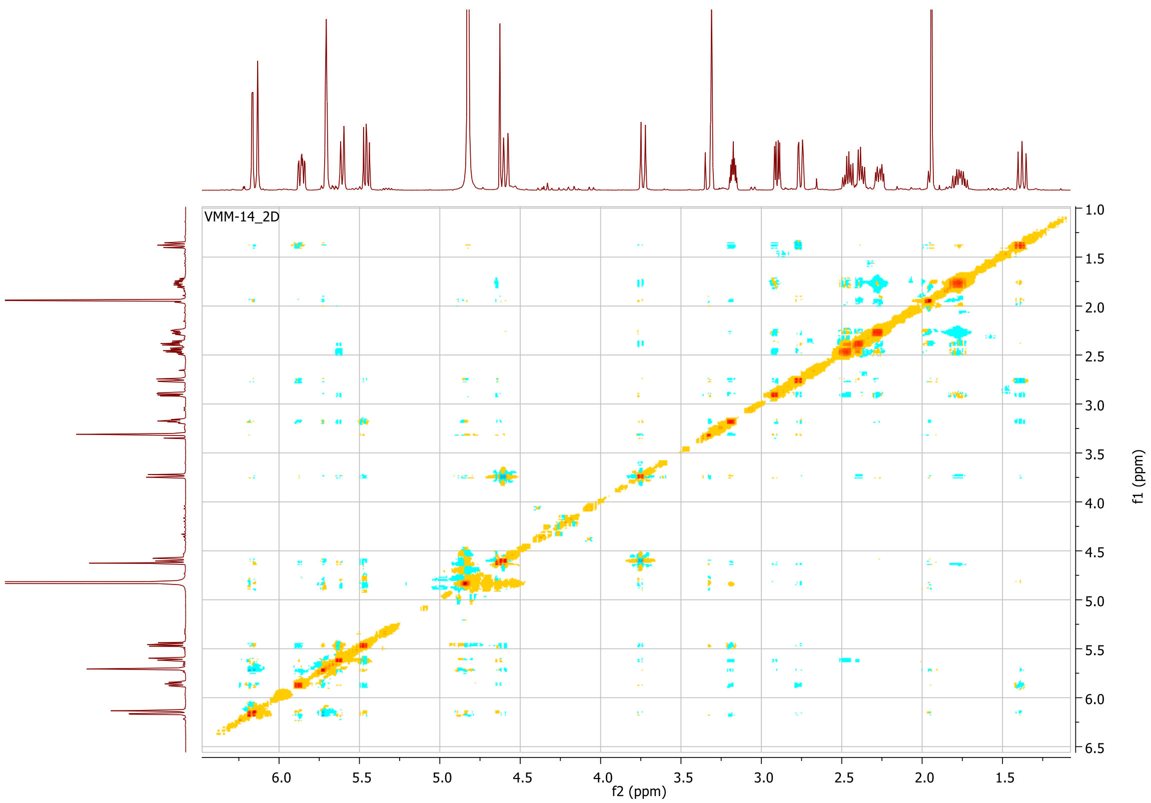

Figure S10. NOESY spectrum of compound **1** (500 MHz, CD_3OD)

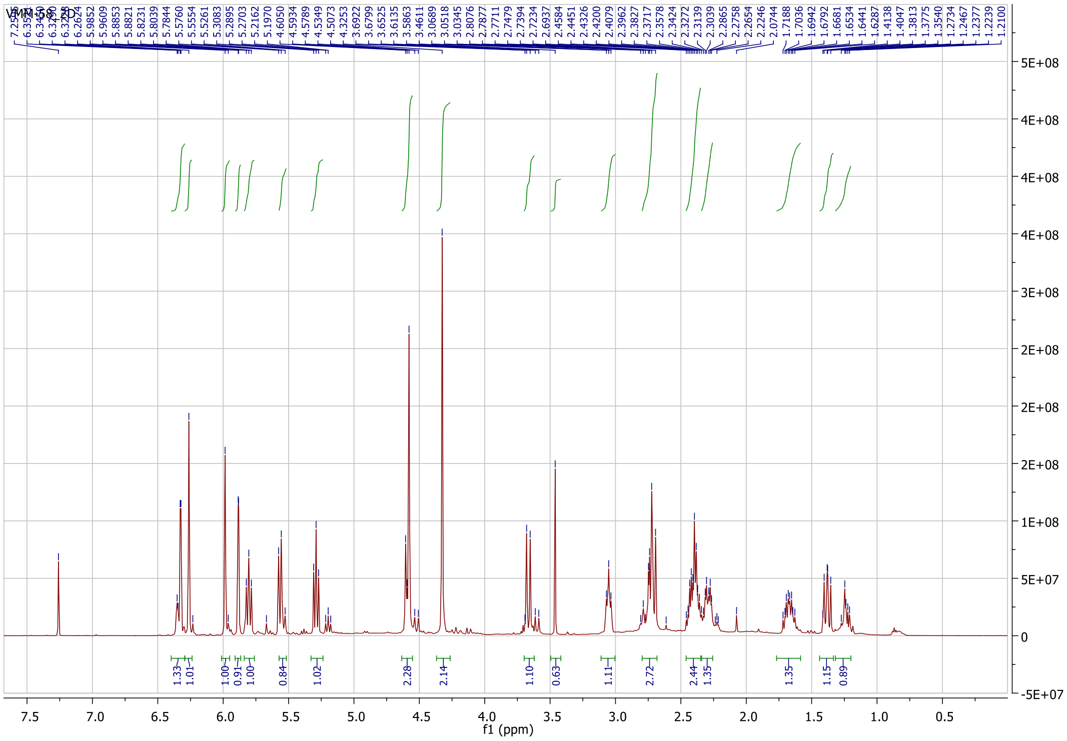

Figure S11. ^1H NMR spectrum of compound **2** (500 MHz, $CDCl_3$)

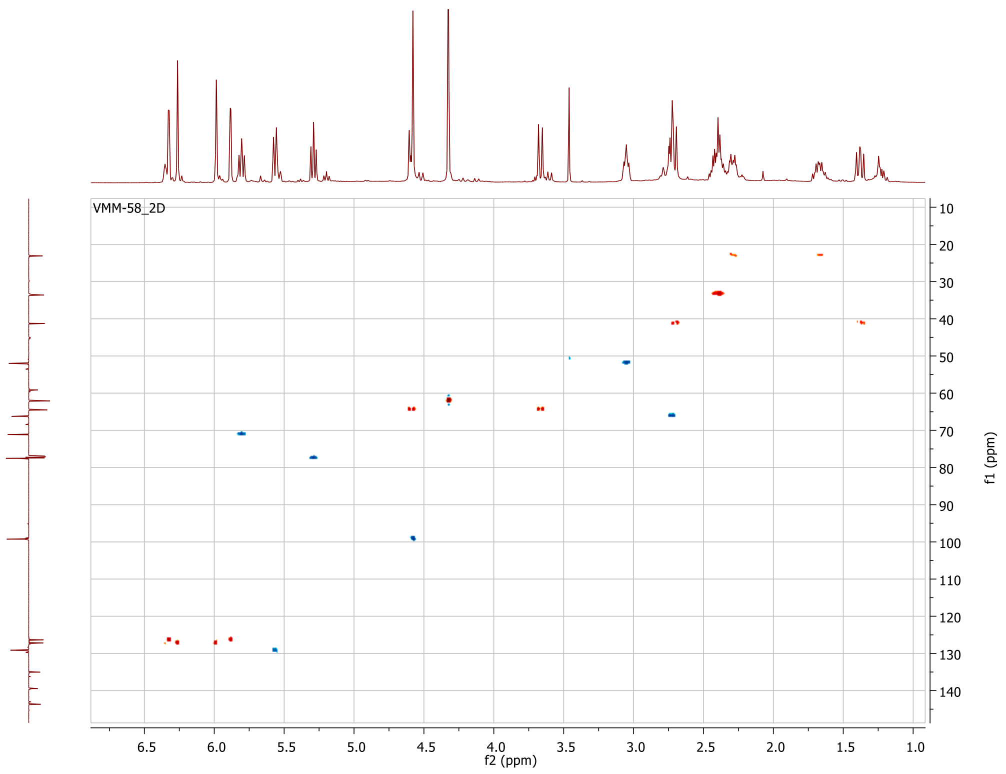

Figure S12. HSQC spectrum of compound **2** (125/500 MHz, $CDCl_3$)

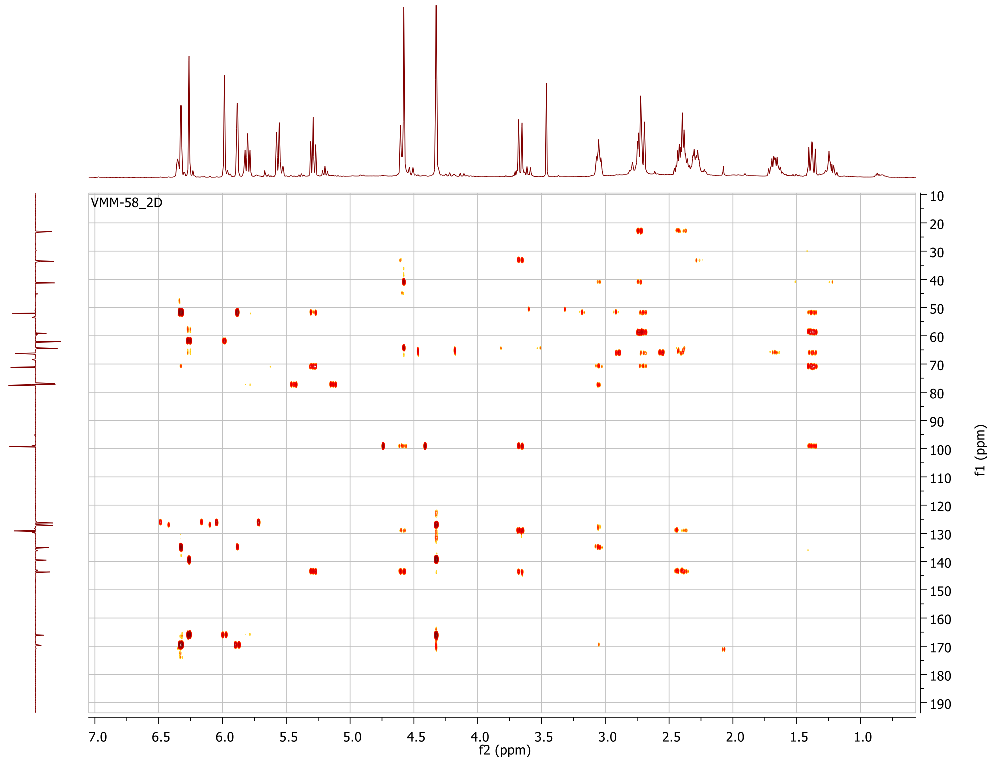

Figure S13. HMBC spectrum of compound **2** (125/500 MHz, $CDCl_3$)

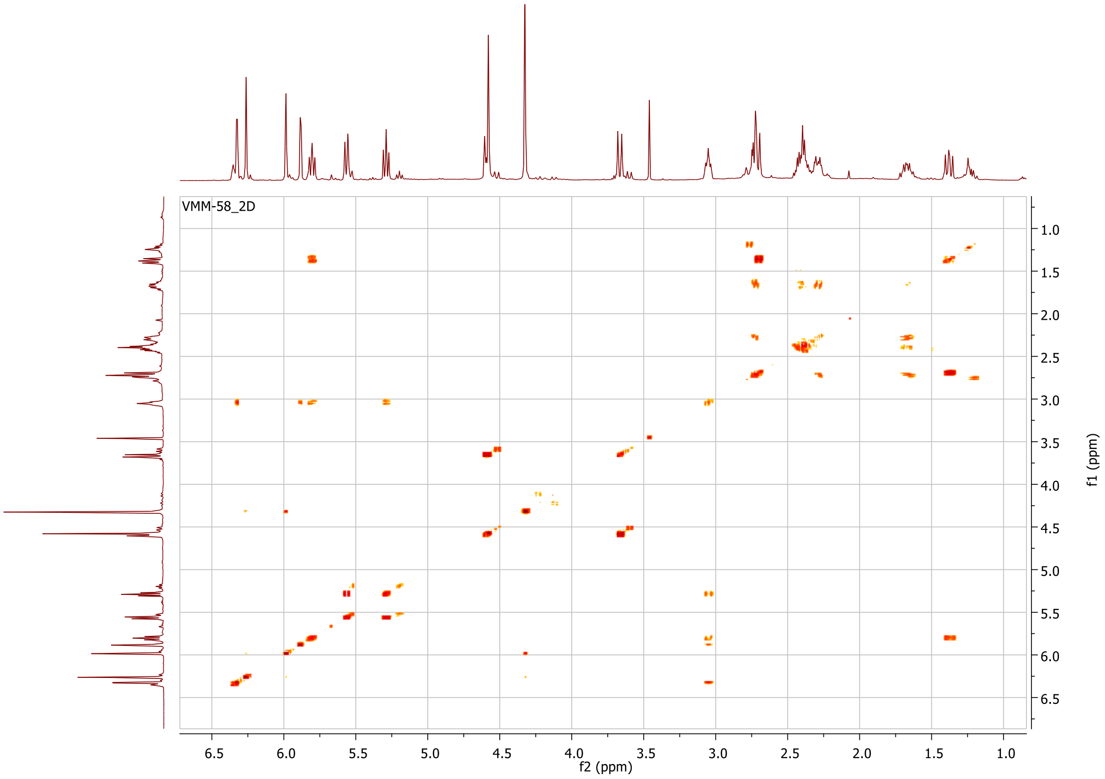

Figure S14. ^{1}H-^{1}H COSY spectrum of compound **2** (500 MHz, $CDCl_3$)

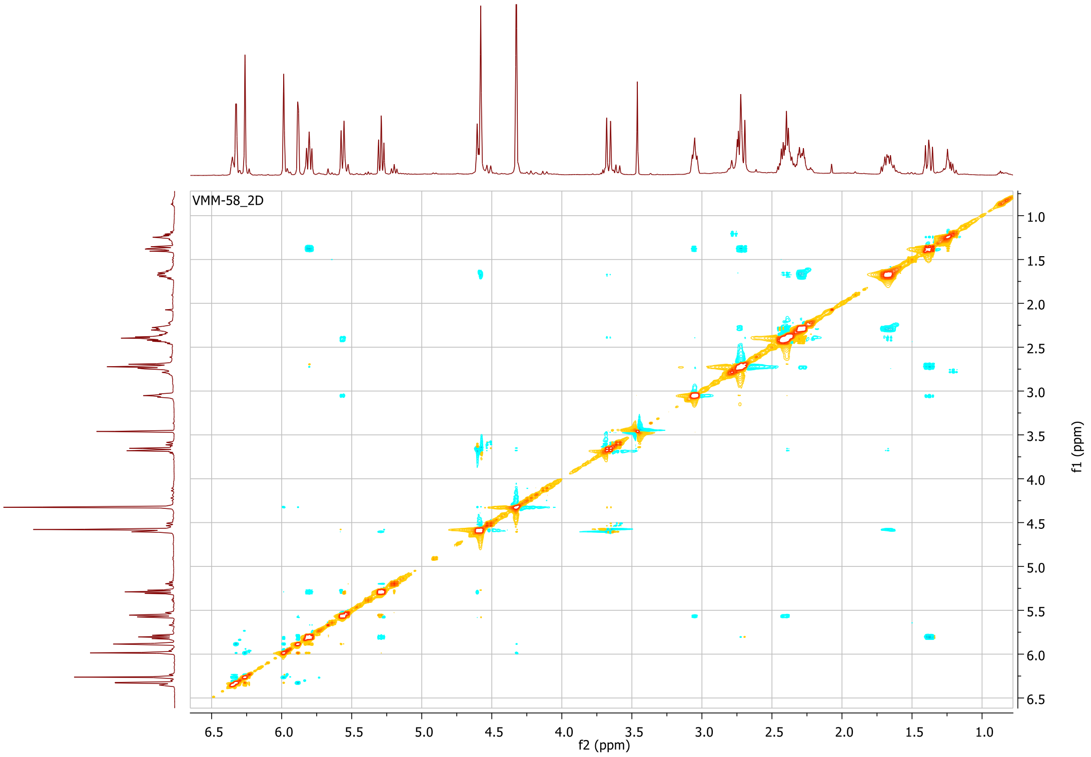

Figure S15. NOESY spectrum of compound **2** (500 MHz, $CDCl_3$)

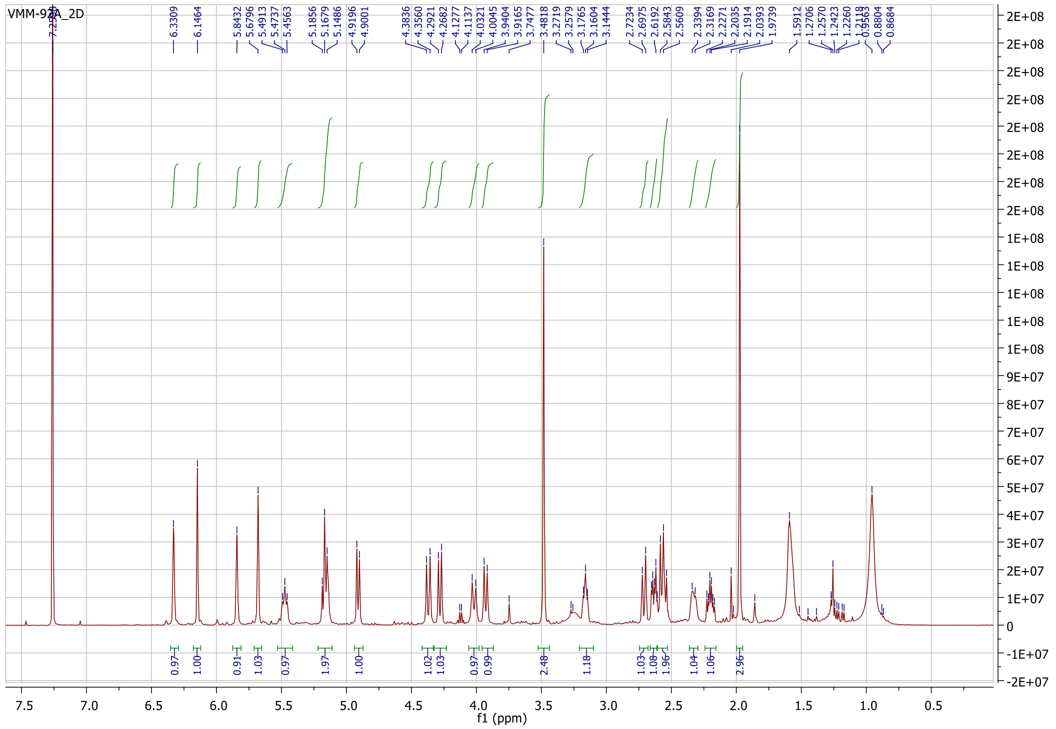

Figure S16. ^{1}H NMR spectrum of compound **3** (500 MHz, $CDCl_3$)

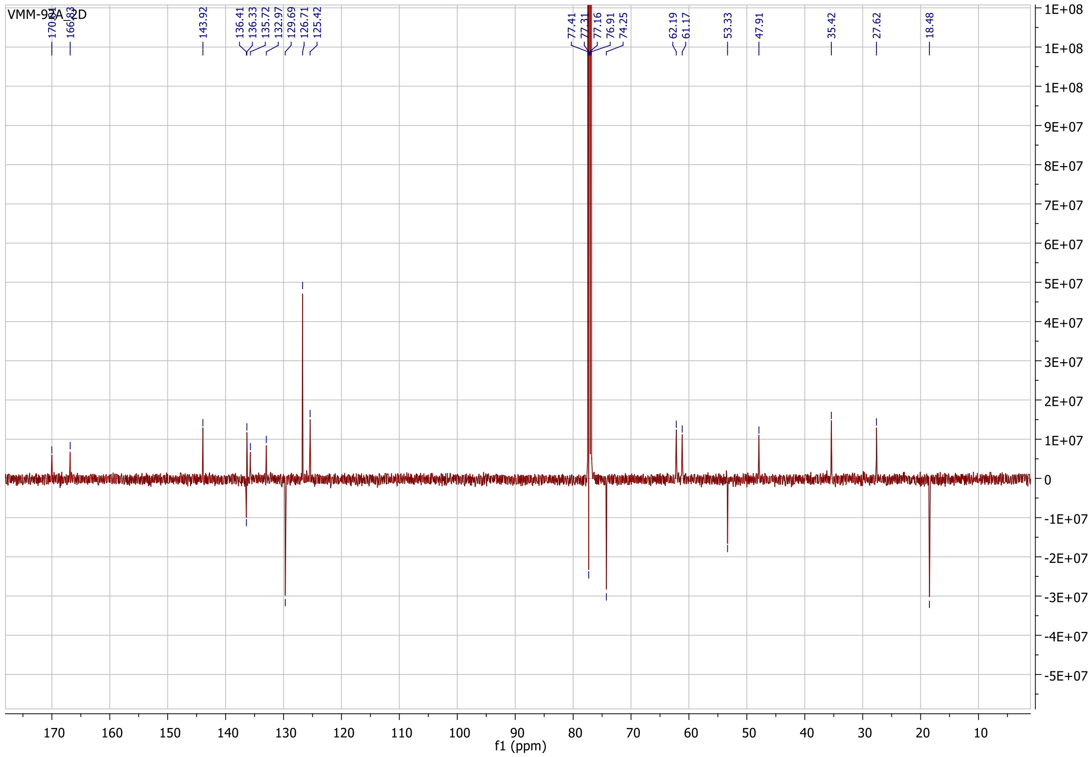

Figure S17. ^{13}C NMR JMOD spectrum of compound **3** (125 MHz, $CDCl_3$)

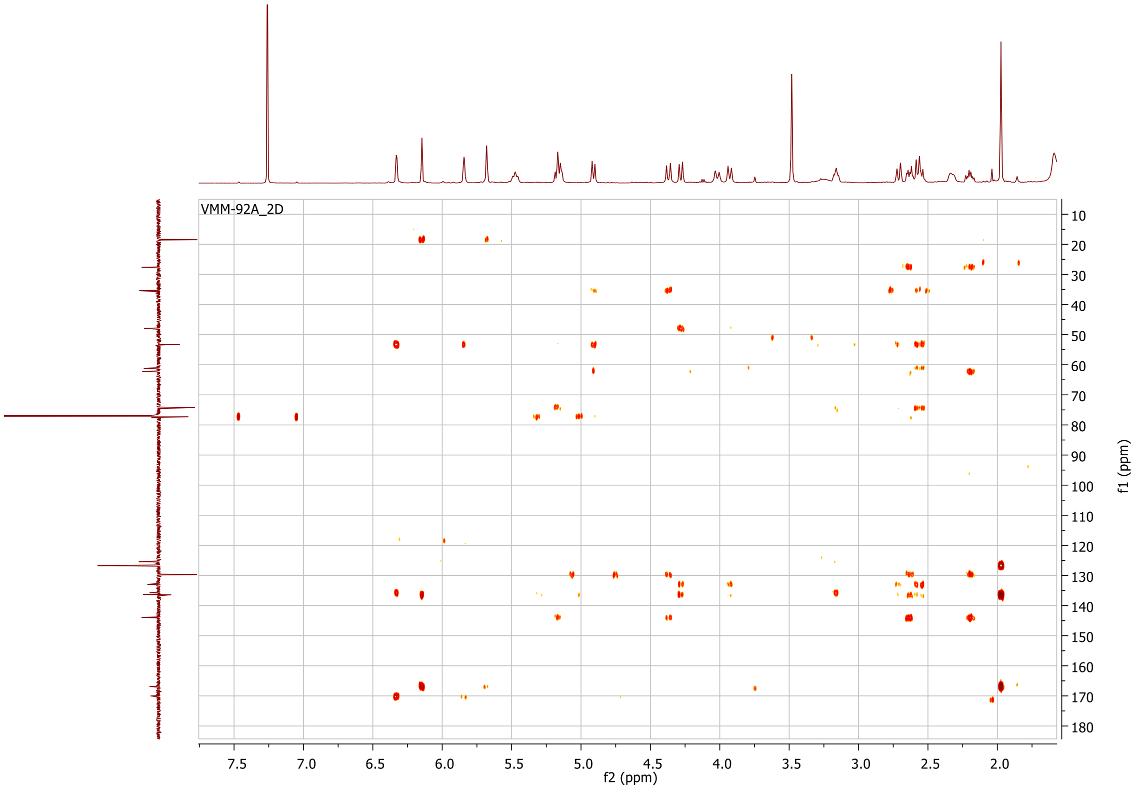

Figure S18. HMBC spectrum of compound **3** (125/500 MHz, $CDCl_3$)

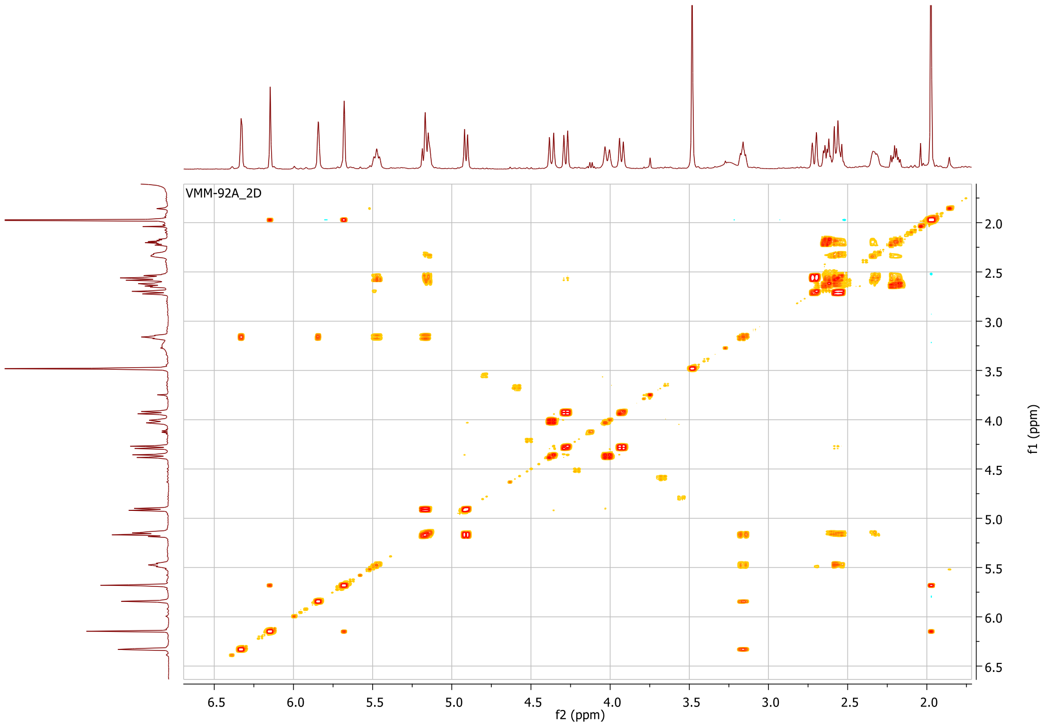

Figure S19. ^1H-^1H COSY spectrum of compound **3** (500 MHz, $CDCl_3$)

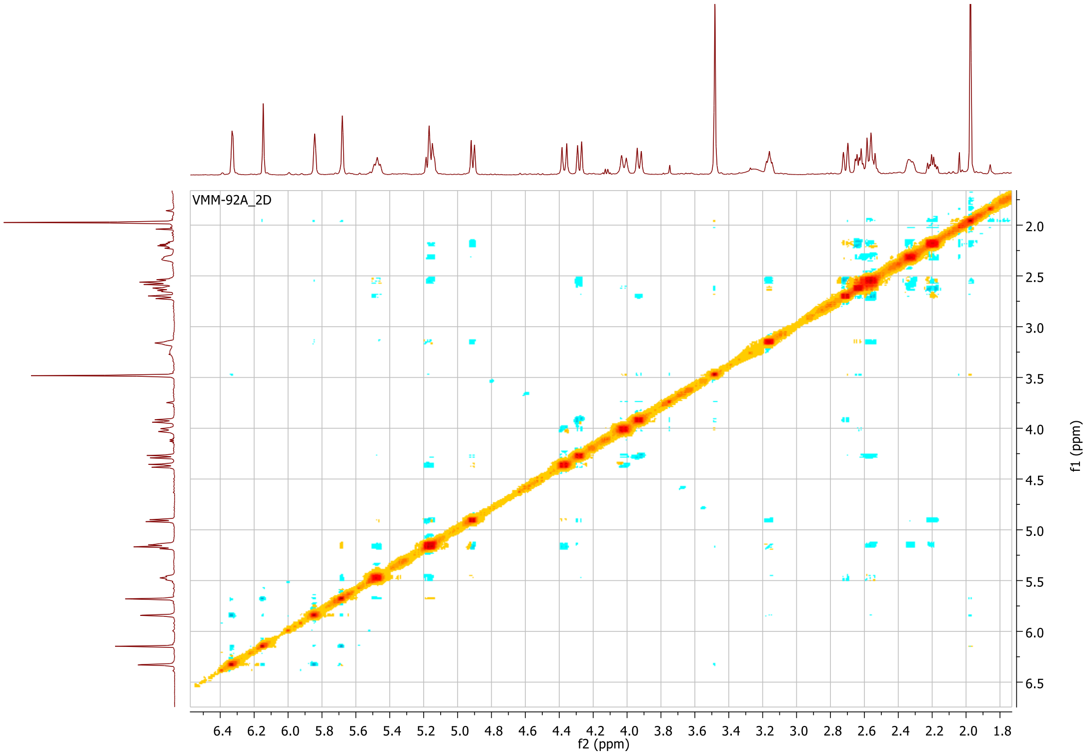

Figure S20. NOESY spectrum of compound **3** (500 MHz, $CDCl_3$)

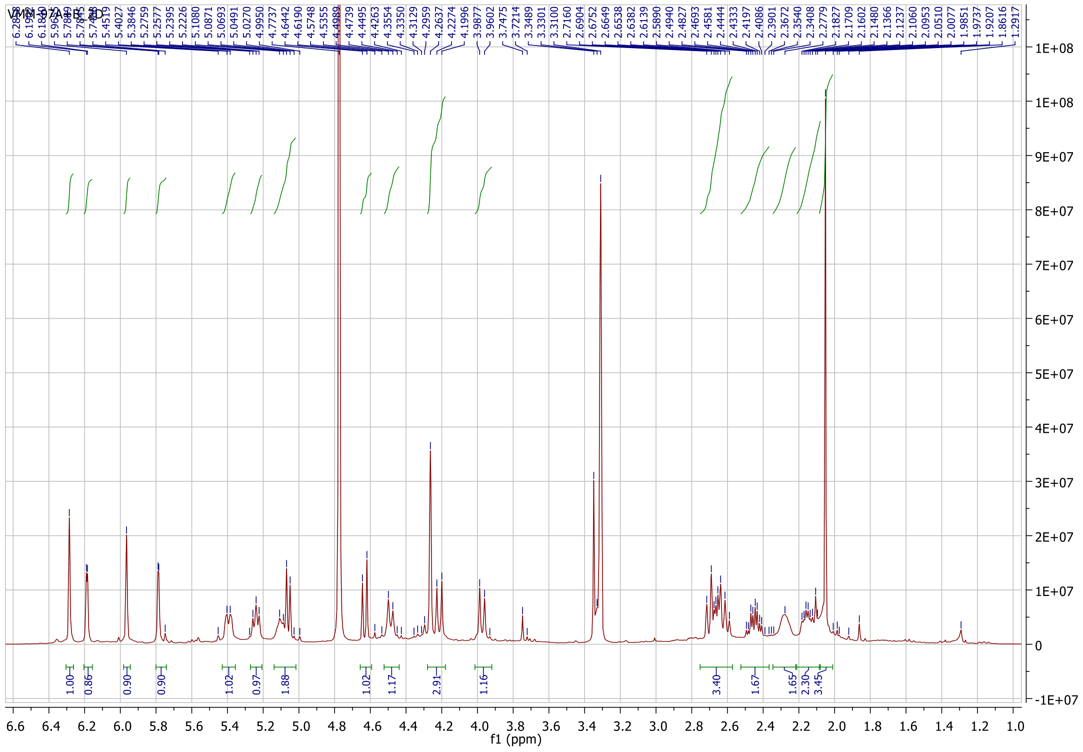

Figure S21. ^1H NMR spectrum of compound **4** (500 MHz, CD_3OD)

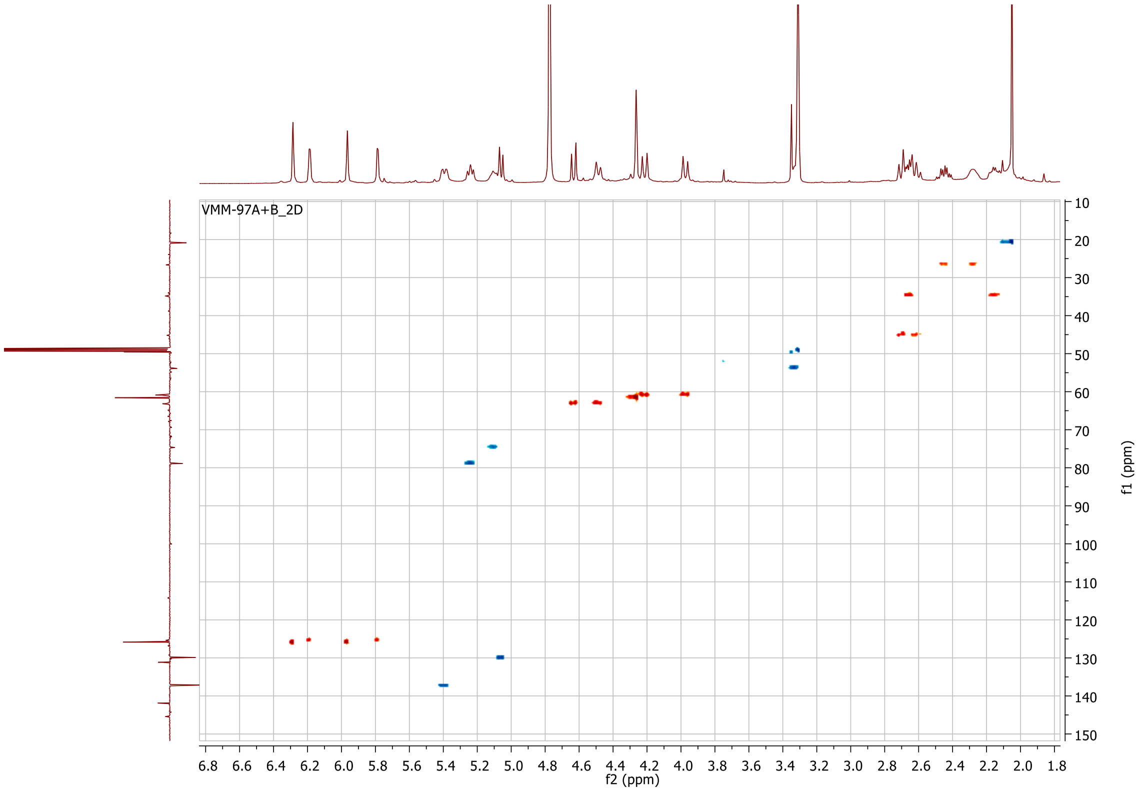

Figure S22. HSQC spectrum of compound **4** (125/500 MHz, CD_3OD)

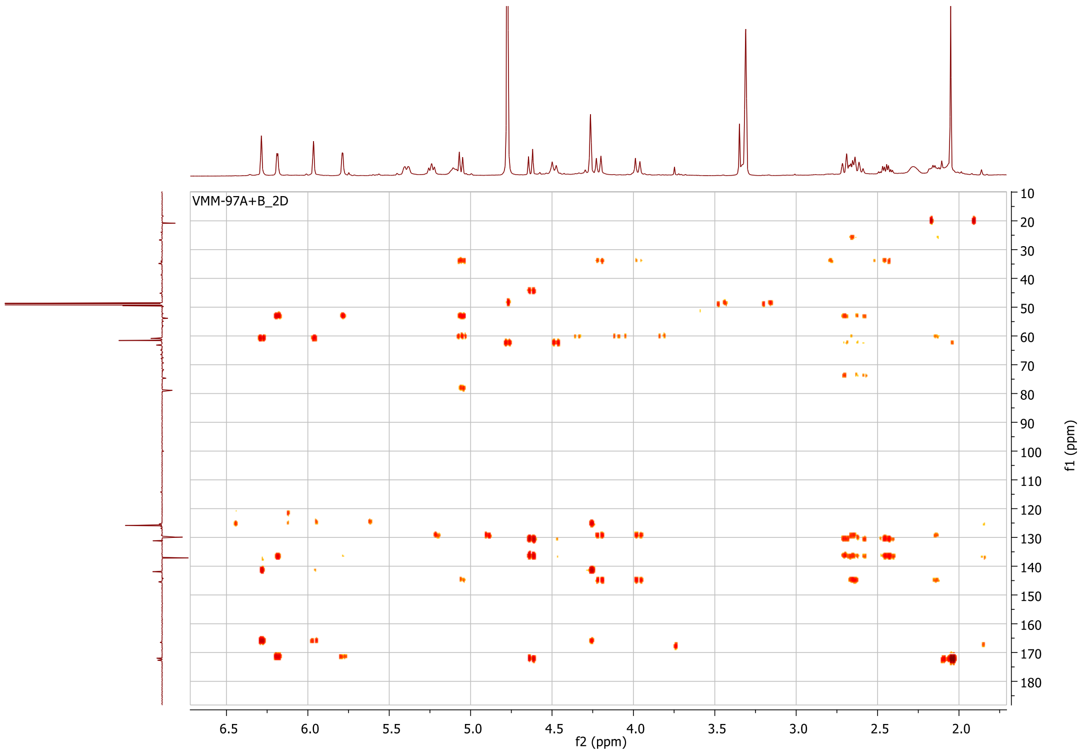

Figure S23. HMBC spectrum of compound **4** (125/500 MHz, CD_3OD)

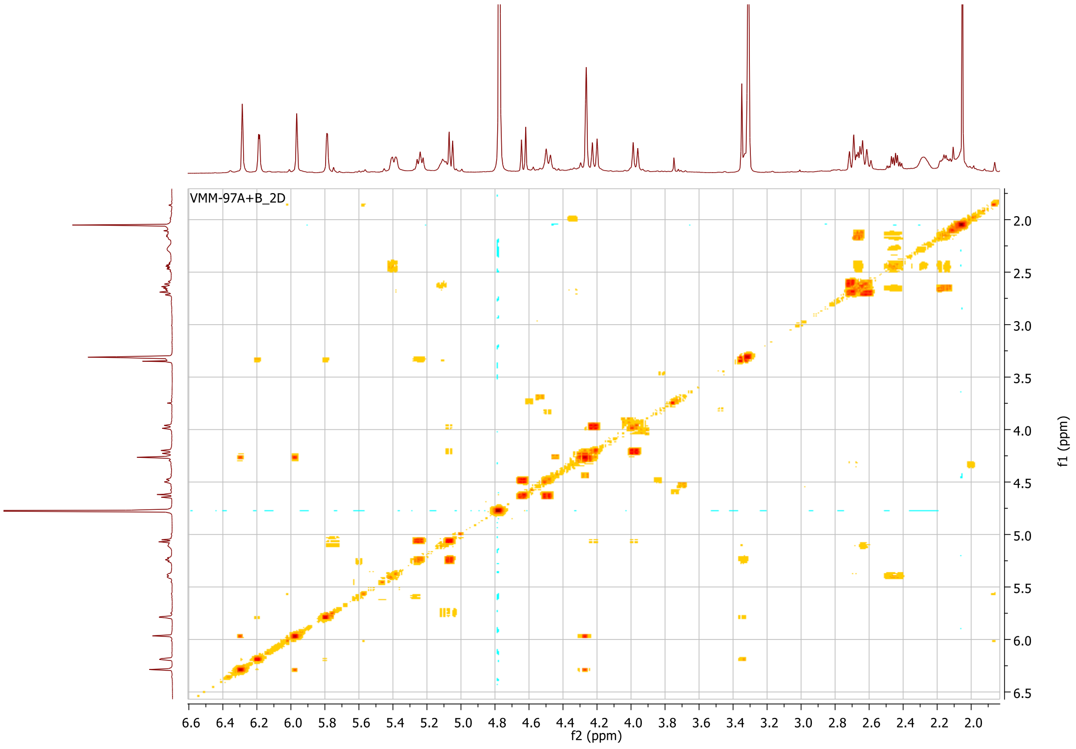

Figure S24. ^{1}H-^{1}H COSY spectrum of compound **4** (500 MHz, CD_3OD)

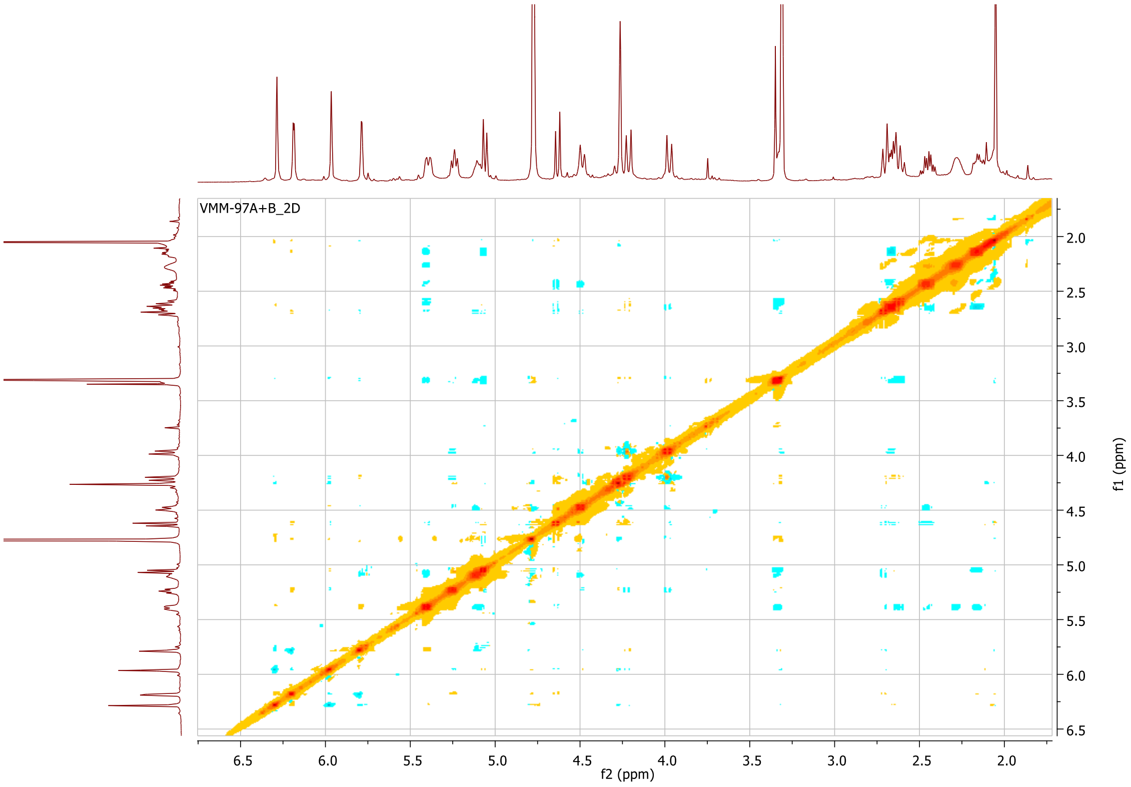

Figure S25. NOESY spectrum of compound **4** (500 MHz, CD_3OD)

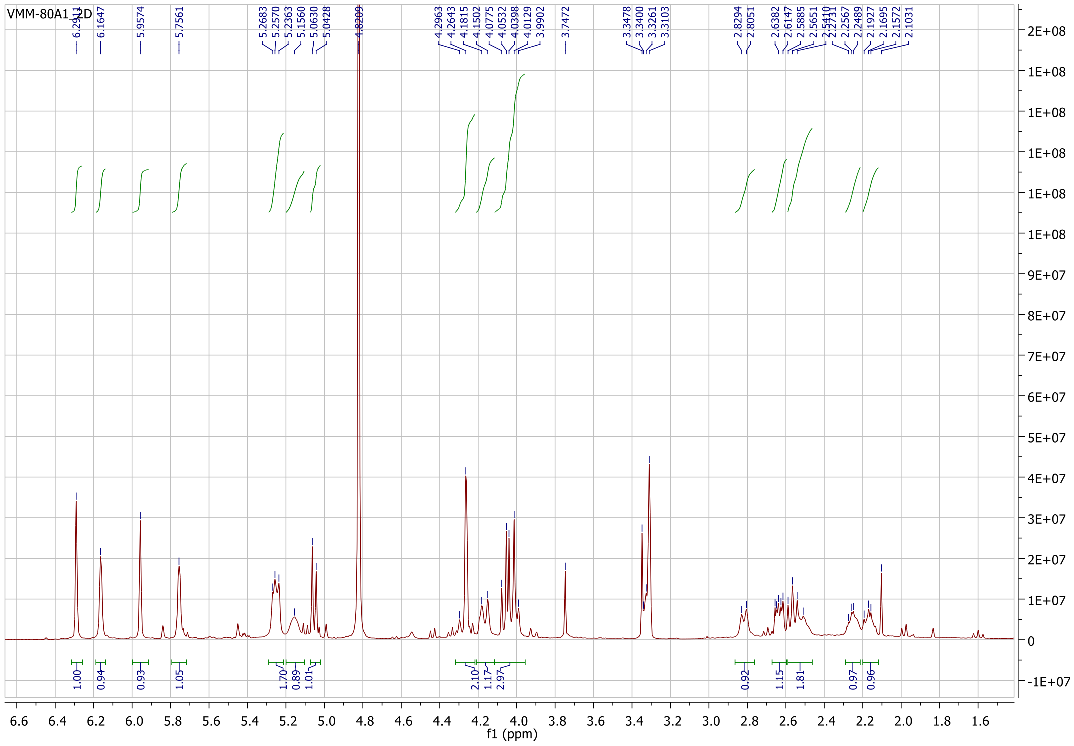

Figure S26. ^{1}H NMR spectrum of compound **5** (500 MHz, CD_3OD)

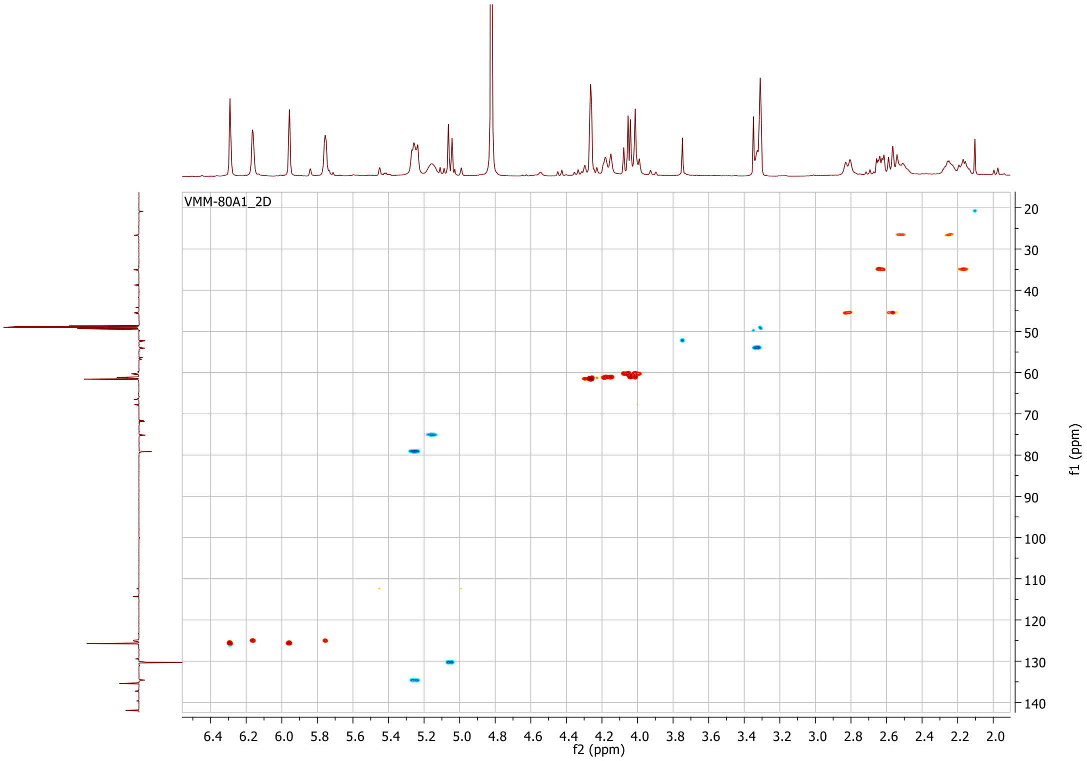

Figure S27. HSQC spectrum of compound **5** (125/500 MHz, CD_3OD)

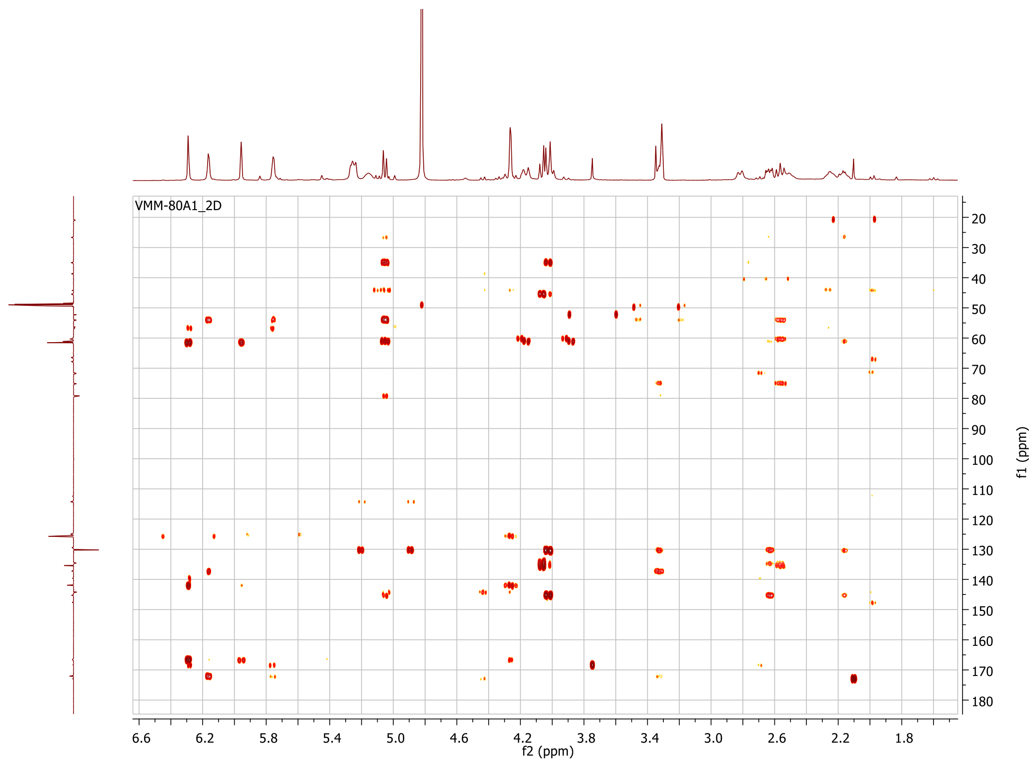

Figure S28. HMBC spectrum of compound **5** (125/500 MHz, CD_3OD)

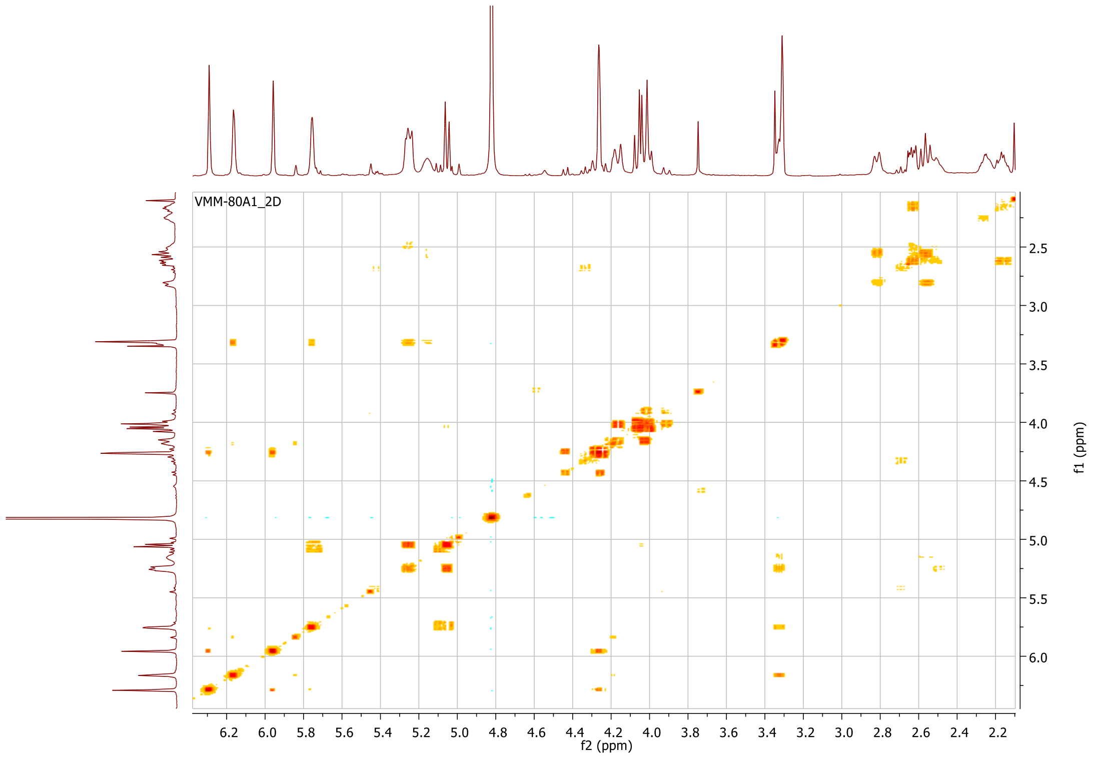

Figure S29. ^1H-^1H COSY spectrum of compound **5** (500 MHz, CD_3OD)

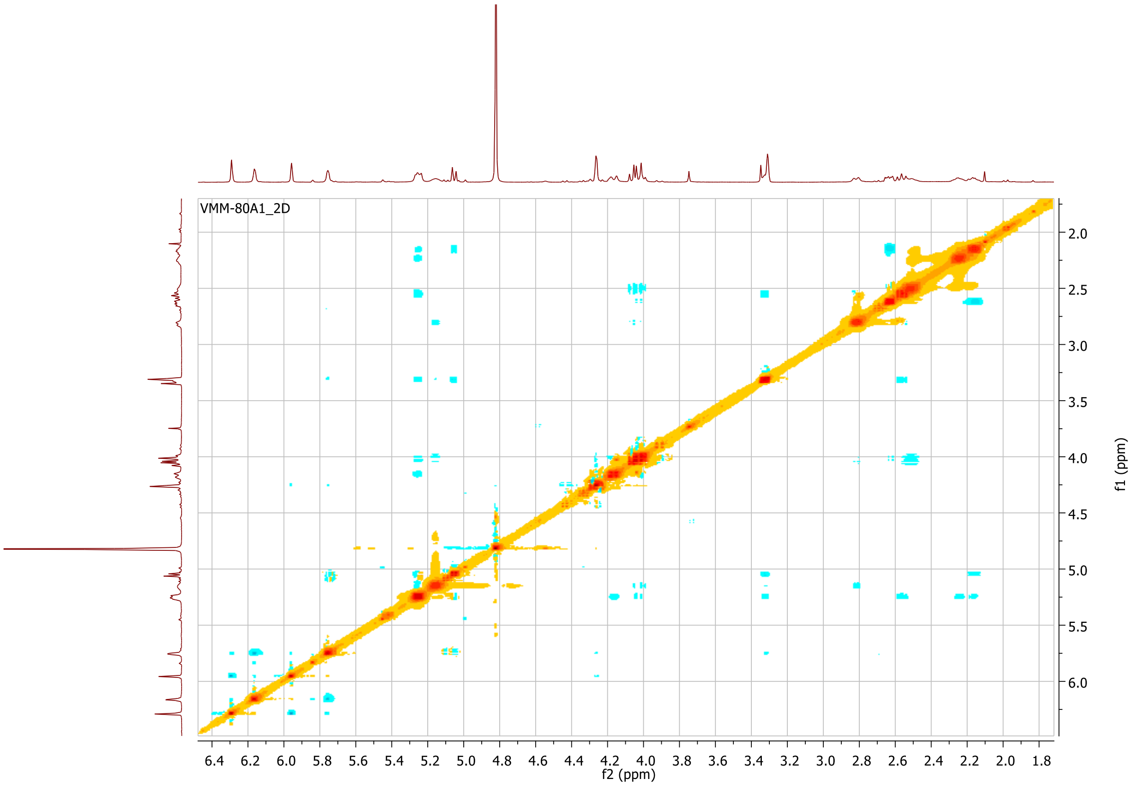

Figure S30. NOESY spectrum of compound **5** (500 MHz, CD_3OD)

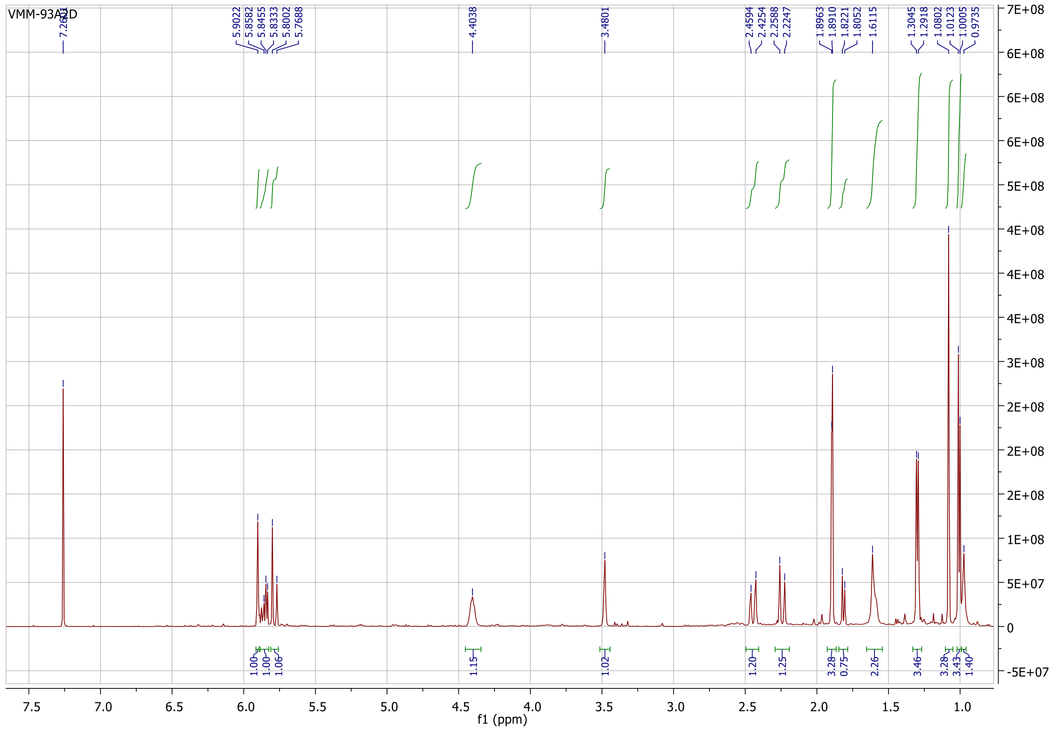

Figure S31. ^1H NMR spectrum of compound **6** (500 MHz, $CDCl_3$)

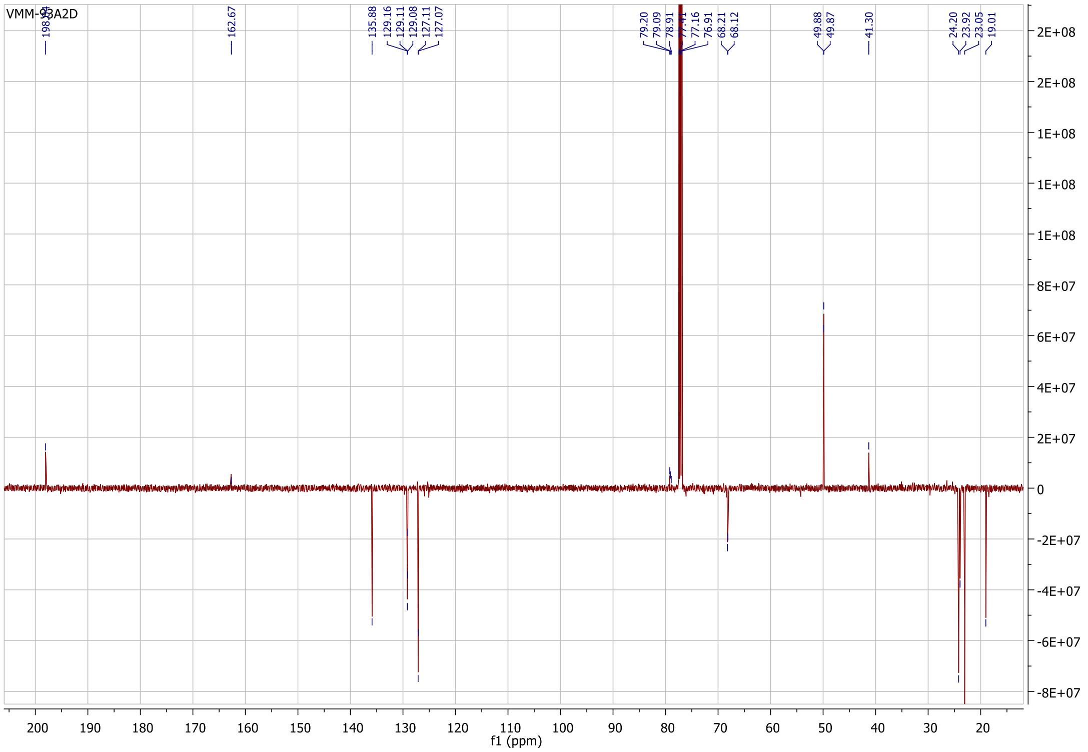

Figure S32. ^{13}C NMR JMOD spectrum of compound **6** (125 MHz, $CDCl_3$)

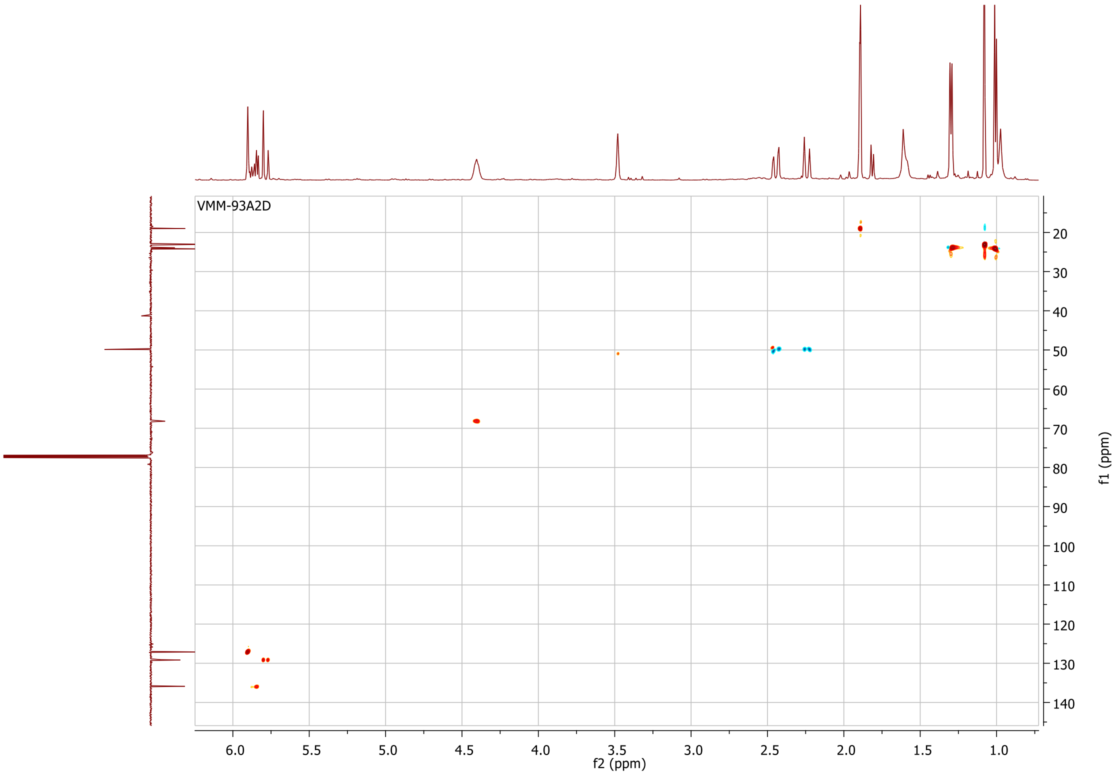

Figure S33. HSQC spectrum of compound **6** (125/500 MHz, $CDCl_3$)

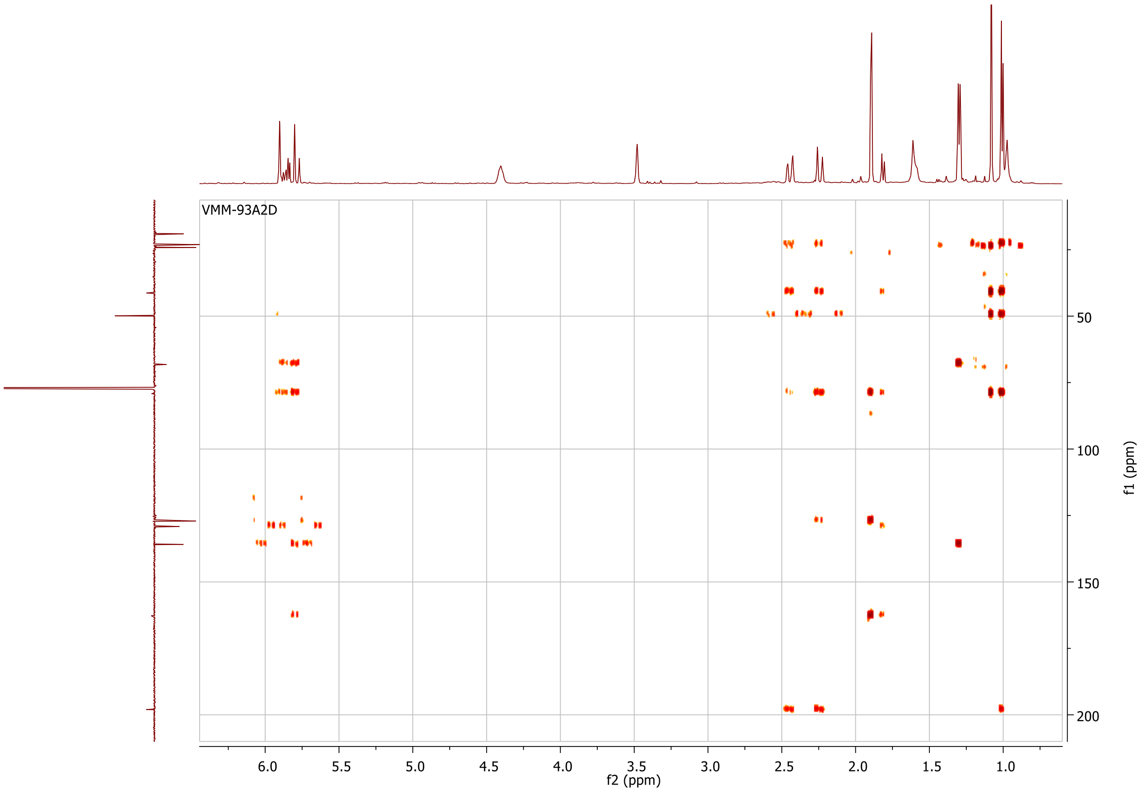

Figure S34. HMBC spectrum of compound **6** (125/500 MHz, $CDCl_3$)

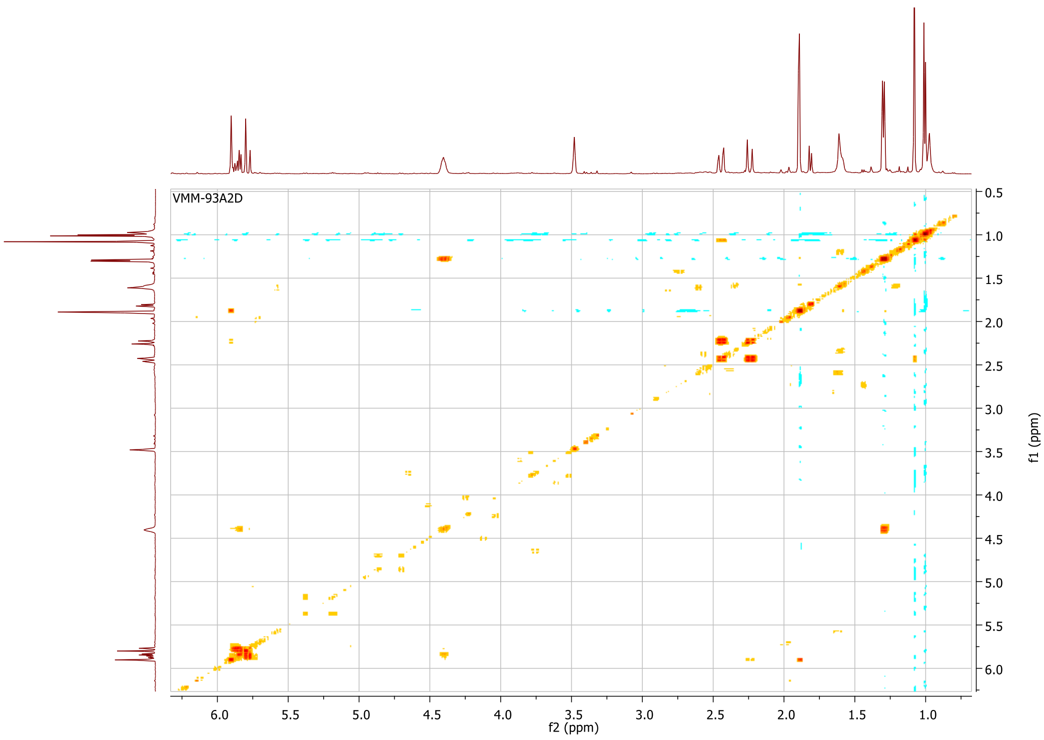

Figure S35. ^1H-^1H COSY spectrum of compound **6** (500 MHz, $CDCl_3$)

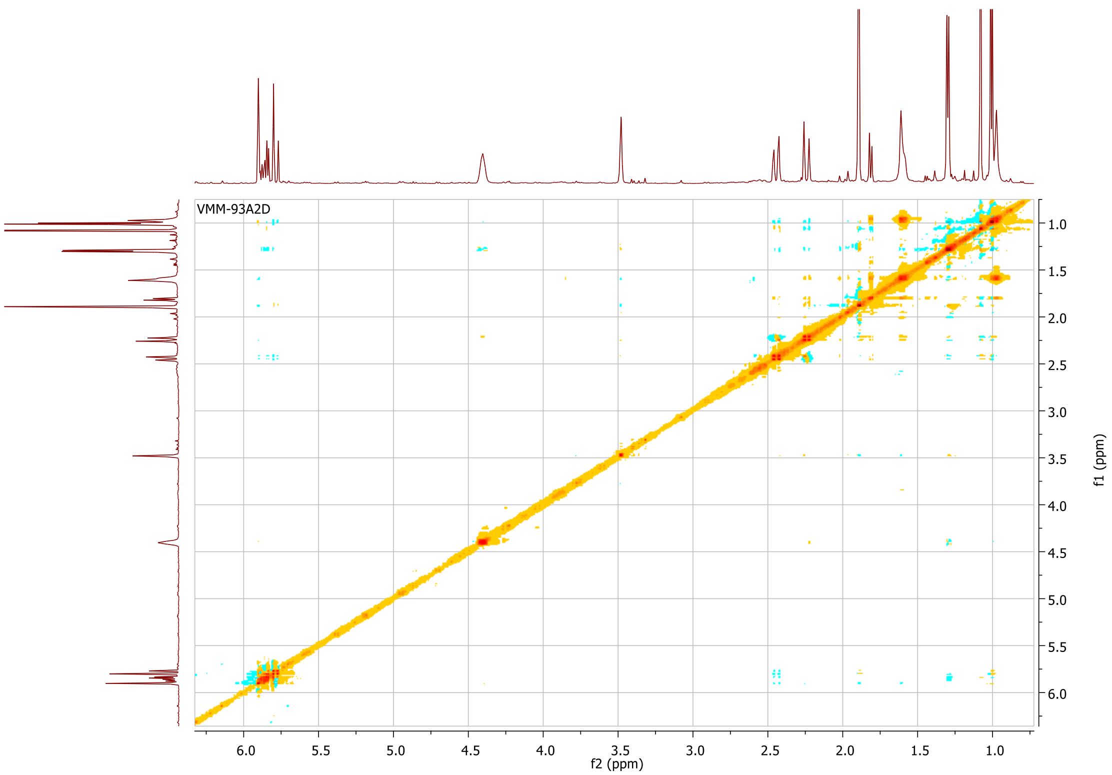

Figure S36. NOESY spectrum of compound **6** (500 MHz, $CDCl_3$)

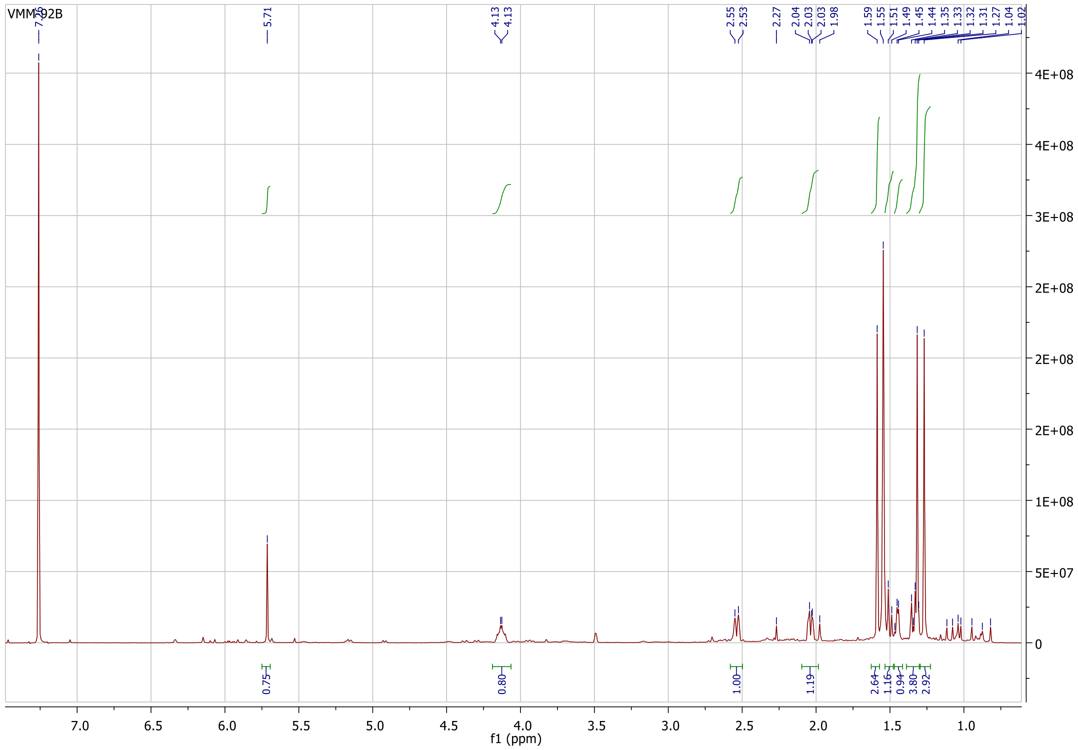

Figure S37. ^1H NMR spectrum of compound **7** (500 MHz, $CDCl_3$)

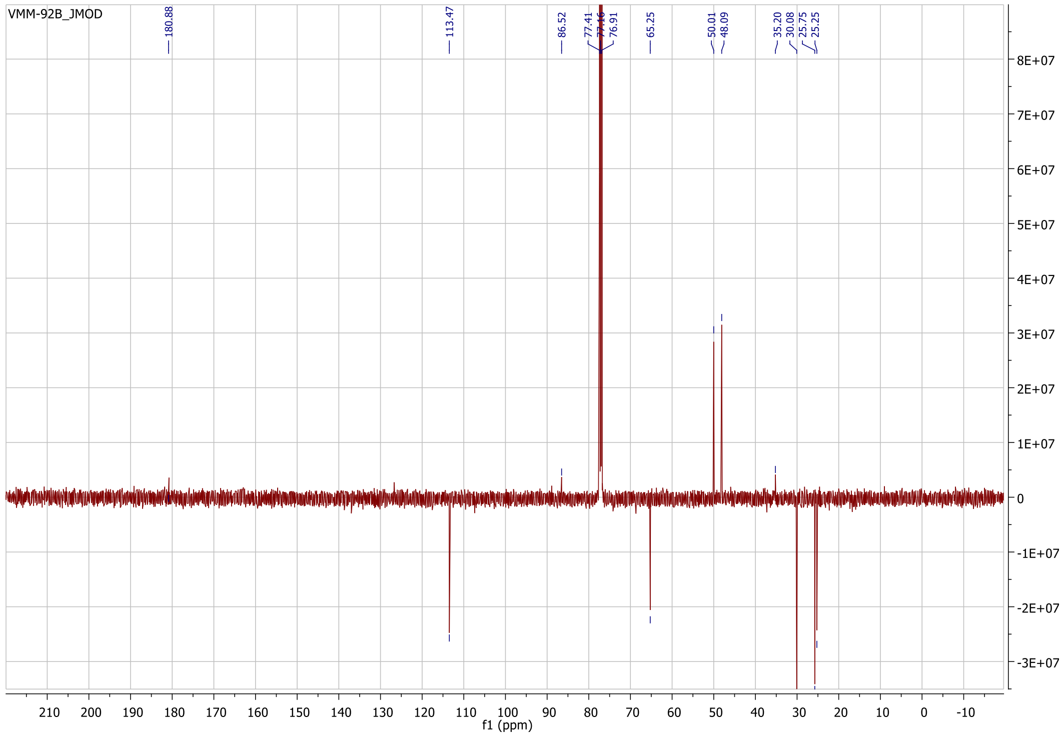

Figure S38. ^{13}C NMR JMOD spectrum of compound **7** (125 MHz, $CDCl_3$)

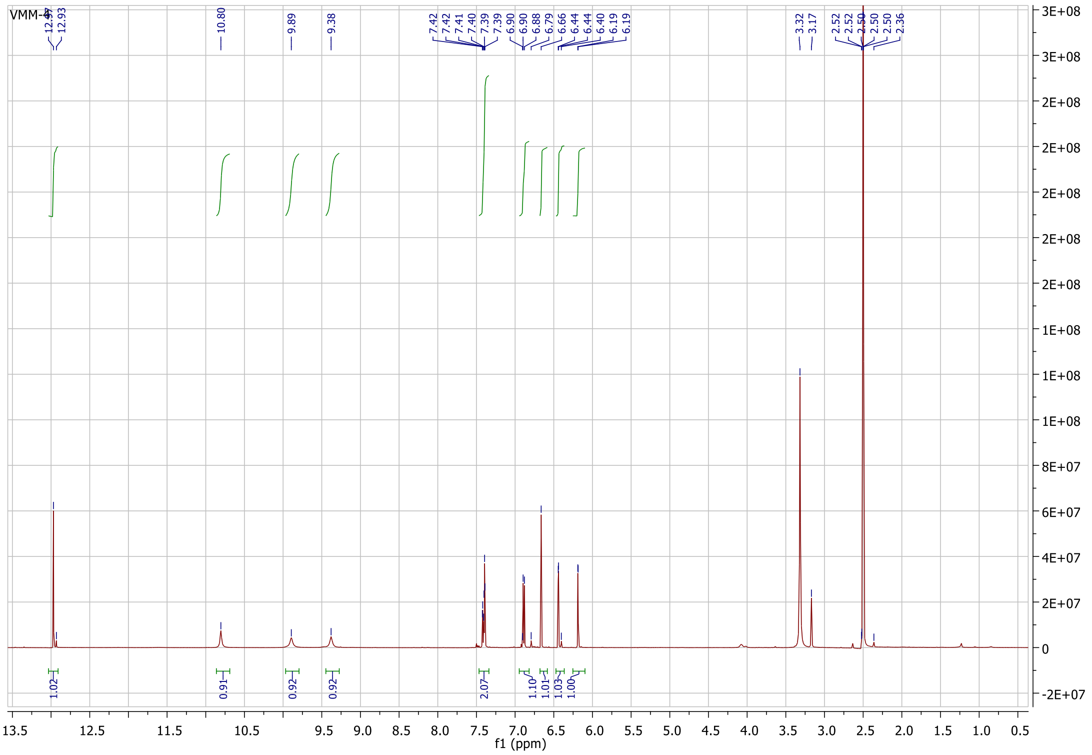

Figure S39. ^{1}H NMR spectrum of compound **8** (500 MHz, DMSO-d_6)

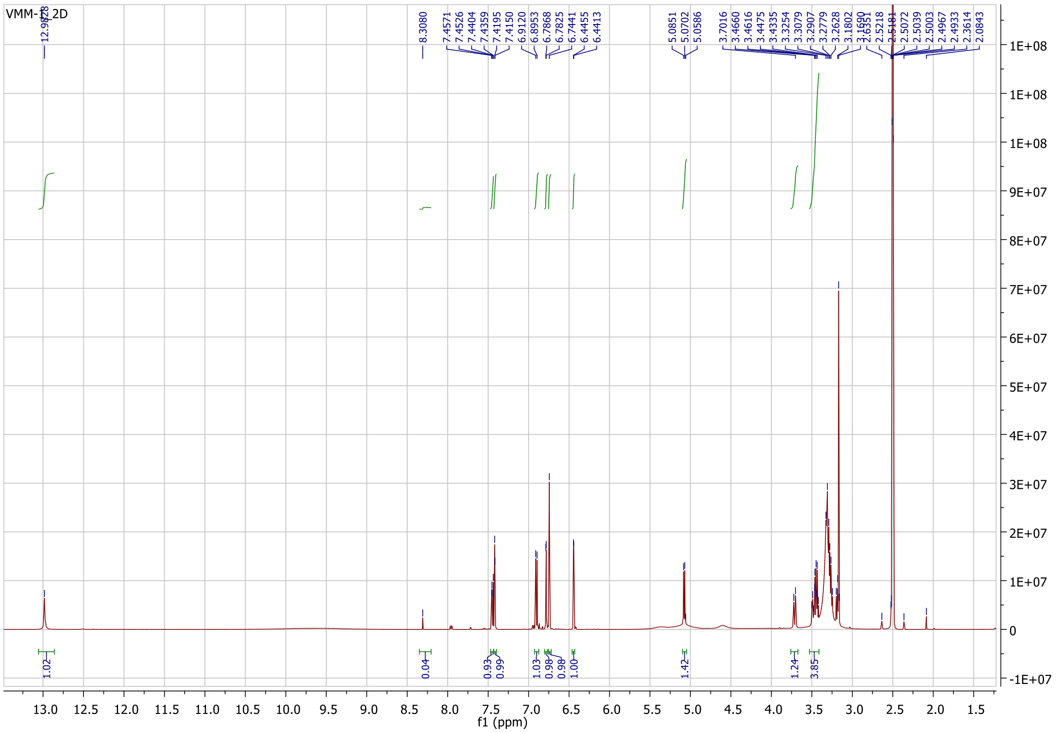

Figure S40. ^{1}H NMR spectrum of compound **9** (500 MHz, DMSO-d_6)

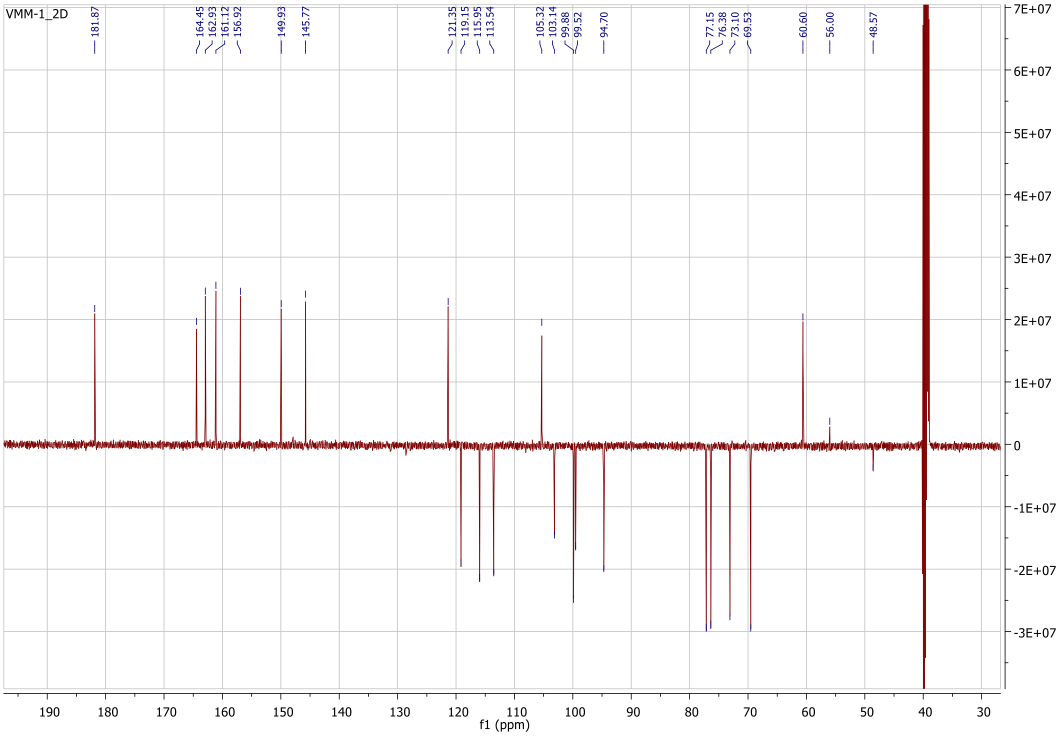

Figure S41. ^{13}C NMR JMOD spectrum of compound **9** (125 MHz, DMSO-d_6)

Table S1. NMR data of vernolide (**1**) and 3'-hydroxyvernolide (**2**) [500 MHz (^{1}H), 125 MHz (^{13}C), δ ppm (J = Hz)]

Position	^{1}H NMR		^{13}C NMR	
	**1**[a]	**2**[b]	**1**[a]	**2**[b]
1	2.90 dd (11.8, 5.0)	2.73 dd (10.4, 5.1)	65.7	66.3
2	1.76 ddt (7.2, 14.0, 11.8)<br>2.26 dt (14.0, 5.0)	1.67 m<br>2.28 dt (13.8, 5.1)	24.7	23.1
3	2.38 dd (12.7, 7.2)<br>2.46 ddd (14.0, 12.7, 7.2)	2.38 m<br>2.42 m	34.3	33.6
4	-	-	144.0	143.7
5	5.61 br d (10.2)	5.57 d (9.8)	131.1	129.1
6	5.46 dd (10.2, 9.8)	5.29 t (9.8)	79.5	77.5
7	3.17 dq (9.8, 5.7)	3.05 m	52.5	52.0
8	5.86 ddd (12.4, 5.7, 2.0)	5.80 t (10.5)	72.2	71.2
9	1.38 t (12.4)<br>2.75 dd (12.4, 2.0)	1.38 dd (14.5, 10.5)<br>2.71 d (14.5)	41.8	41.3
10	-	-	60.5	59.1
11	-	-	137.4	134.0
12	-	-	171.8	169.7
13	5.71 d (4.1)<br>6.17 d (4.1)	5.88 d (2.3)<br>6.33 d (2.3)	125.5	126.3
14	4.63 s	4.58 s	100.0	99.3
15	3.74 dd (13.7, 1.0)<br>4.59 d (13.7)	3.66 d (13.9)<br>4.59 d (13.9)	64.5	64.5
1'	-	-	167.6	166.1
2'	-	-	137.8	139.5
3'	5.70 s<br>6.13 s	5.99 s<br>6.26 s	126.9	127.2
4'	1.94 s	4.33 s (2H)	18.4	62.1

[a] in CD_3OD; [b] in $CDCl_3$

Table S2. NMR data of pectorolide (**3**) and 4’-hydroxypectorolide (**5**) [500 MHz (^{1}H), 125 MHz (^{13}C), δ ppm (J = Hz)]

**Position**	**^{1}H**		**^{13}C**	
	**3**[a]	**5**[b]	**3**[a]	**5**[b]
1	5.15 m	5.26 m	136.4	134.6
2	2.33 m<br>2.56 m	2.25 m<br>2.51 m	27.6	26.7
3	2.20 ddd (12.5, 11.8, 6.3)<br>2.64 dd (12.5, 5.8)	2.17 m<br>2.64 dd (11.2, 5.8)	35.4	35.0
4	-	-	143.9	145.2
5	4.91 d (9.8)	5.05 d (10.3)	129.7	130.3
6	5.17 t (8.7)	5.26 dd (10.3, 8.9)	77.3	79.2
7	3.16 t (8.1)	3.33 t (8.9)	53.3	54.0
8	5.47 t (9.1)	5.16 br s	74.3	75.2
9	2.56 dd (12.5, 9.1)<br>2.71 d (12.5)	2.57 t (12.1)<br>2.82 d (12.1)	47.9	45.5
10	-	-	133.0	135.4
11	-	-	135.7	137.3
12	-	-	170.0	172.1
13	5.84 s<br>6.33 s	5.76 s<br>6.16 s	125.4	125.1
14	3.93 d (12.0)<br>4.28 d (12.0)	4.00 d (11.5)<br>4.07 d (11.5)	61.2	60.3
15	4.02 d (13.8)<br>4.37 d (13.8)	4.03 d (14.6)<br>4.16 d (14.6)	62.2	61.1
1’	-	-	166.8	166.7
2’	-	-	136.3	141.9
3’	5.68 s<br>6.15 s	5.95 s<br>6.29 s	126.7	125.7
4’	1.97 s	4.26 s (2H)	18.5	61.6

[a] in $CDCl_3$; [b] in CD_3OD
